# MiR-135 suppresses glycolysis and promotes pancreatic cancer cell adaptation to metabolic stress by targeting phosphofructokinase-1

**DOI:** 10.1038/s41467-019-08759-0

**Published:** 2019-02-18

**Authors:** Ying Yang, Mari B. Ishak Gabra, Eric A. Hanse, Xazmin H. Lowman, Thai Q. Tran, Haiqing Li, Neta Milman, Juan Liu, Michael A. Reid, Jason W. Locasale, Ziv Gil, Mei Kong

**Affiliations:** 1 Department of Molecular Biology and Biochemistry, School of Biological Sciences, University of California, Irvine, CA 92697 USA; 2 Center for Informatics, City of Hope, Duarte, CA 91010 USA; 3 Department of Computational & Quantitative Medicine, Beckman Research Institute, City of Hope, Duarte, CA 91010 USA; 4 Laboratory for Applied Cancer Research, Department of Otolaryngology Head and Neck Surgery, Rambam Healthcare Campus, The Technion-Israel Institute of Technology, Haifa, 3109601 Israel; 5 Department of Pharmacology and Cancer Biology, Duke University School of Medicine, Durham, NC 27708 USA

## Abstract

Pancreatic ductal adenocarcinoma (PDAC) is one of the most lethal human cancers. It thrives in a nutrient-poor environment; however, the mechanisms by which PDAC cells undergo metabolic reprogramming to adapt to metabolic stress are still poorly understood. Here, we show that microRNA-135 is significantly increased in PDAC patient samples compared to adjacent normal tissue. Mechanistically, miR-135 accumulates specifically in response to glutamine deprivation and requires ROS-dependent activation of mutant p53, which directly promotes miR-135 expression. Functionally, we found miR-135 targets phosphofructokinase-1 (PFK1) and inhibits aerobic glycolysis, thereby promoting the utilization of glucose to support the tricarboxylic acid (TCA) cycle. Consistently, miR-135 silencing sensitizes PDAC cells to glutamine deprivation and represses tumor growth in vivo. Together, these results identify a mechanism used by PDAC cells to survive the nutrient-poor tumor microenvironment, and also provide insight regarding the role of mutant p53 and miRNA in pancreatic cancer cell adaptation to metabolic stresses.

## Abstract (web summary)

Pancreatic ductal adenocarcinoma must adapt to a nutrient-poor microenvironment. Here, the authors show that miR-135 accumulates in response to glutamine deprivation and inhibits aerobic glycolysis by targeting phosphofructokinase-1, thereby redirecting glucose carbon to the TCA cycle and allowing pancreatic cancer cells survival.

## Introduction

Pancreatic ductal adenocarcinoma (PDAC) is the fourth leading cause of cancer deaths in the United States, with a 5-year survival rate of 8%^1^. Since the pancreas has an anatomically inaccessible location that prevents routine examination^2^, this low survival rate is largely attributed to advanced stages diagnosis, when PDAC patients already exhibit metastasis; therefore, surgical or chemotherapeutic interventions have minimal impact^3,4^. Consequently, early-stage detection methods and effective preventive strategies are urgently needed for improving the death rates of this disease^4^. One obstacle underlying these clinical challenges is our limited understanding of how PDAC reprograms metabolism in the unique tumor microenvironment^5^. Unlike the more extensive understanding of the mutational mechanisms that initiate PDAC, the metabolic rewiring in this disease is still unclear.

Compared to other cancer types, PDAC is unique due to the notable extent of its desmoplastic reaction, which often forms dense stroma^6–8^. This dense tumor mass in PDAC leads to the generation of high levels of solid stress and fluid pressure in the tumors and compression of the vasculature, thereby creating a highly hypoxic and nutrient-poor microenvironment^9–12^. Thus, the lack of nutrients imposes major challenges for cells to maintain redox and metabolic homeostasis, as well as minimal support for macromolecular biosynthesis, which indicates that PDAC cells may reprogram metabolic pathways to support different energetic and biosynthetic demands in a state of constant nutrient deprivation^10,13,14^.

MicroRNAs, a class of 18−23 nucleotide noncoding RNAs, have gained much attention as a new family of molecules involved in mediating metabolic stress response in cancer^15,16^. For example, miRNAs can modulate critical signaling pathways such as LKB1/AMPK^16^, p53^17^, c-Myc^18^, PPARδ^19^, and ISCU1/2^20^ that regulate metabolism indirectly. In this study, using RNA-seq analysis, we find miR-135b is upregulated in pancreatic cancer patient samples which is consistent with the report that miR-135b is a reported biomarker in pancreatic cancer patients^21^. Yet, the function of miR-135b in PDAC is unknown.

Here, compared to other metabolic stress, we show that both miR-135a and miR-135b are induced specifically under low glutamine conditions and are essential for PDAC cell survival upon glutamine deprivation in vitro and in vivo. We further demonstrate PFK1, a critical enzyme for glycolytic flux, is a miR-135 family target gene. Using metabolic tracer-labeling experiments, we show that miR-135 expression suppresses aerobic glycolysis and promotes glucose carbon contribution to the tricarboxylic acid (TCA) cycle, thus decreasing the glutamine dependence of PDAC cells. Consistently, we find PDAC patients express decreased PFK1 expression with inversely correlative higher levels of miR-135. This study delineates a previously unidentified pathway, in which PDAC senses glutamine levels and provides important evidence that miRNA is actively involved in pancreatic cancer cell adaptation to the nutrient-poor microenvironment.

## Results

### miR-135 is induced upon glutamine deprivation in PDAC cells

**Fig. 1:**
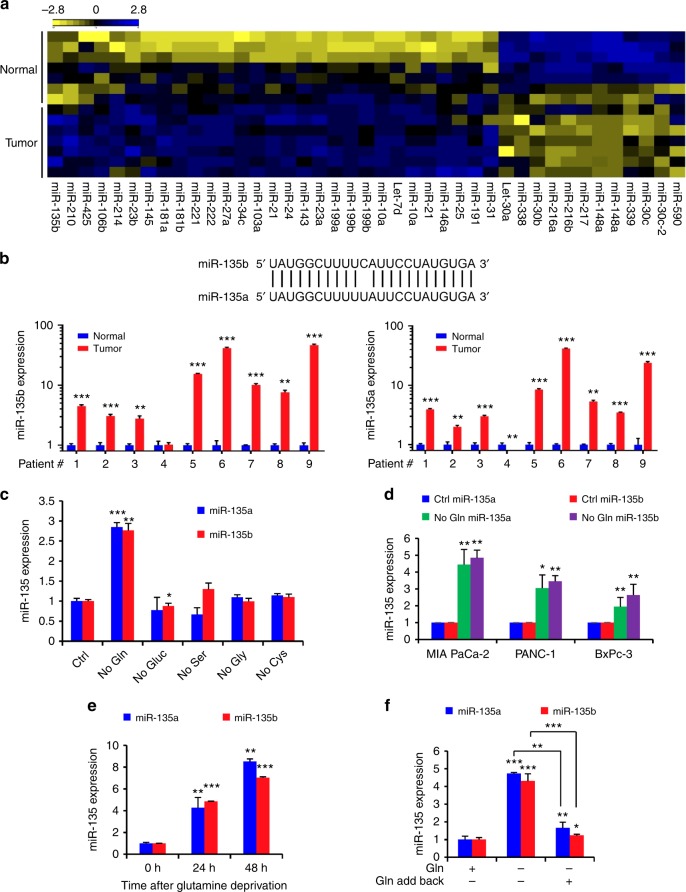
miR-135 is induced upon glutamine deprivation in PDAC cells. **a** Heatmap of miRNAs expression in human pancreatic tumors compared with normal tissues measured by RNA-seq. **b** Alignment between mature miR-135b and miR-135a indicating one nucleotide difference; miR-135b and miR-135a expression in nine pairs of pancreatic tumors compared with adjacent normal tissues were measured by qPCR. Each value represents the mean ± SD in three independent experiments. **p < 0.01, ***p < 0.001, Student’s *t* test. **c** MIA PaCa-2 cells were cultured in medium without glutamine, glucose, serine, glycine or cysteine for 24 h. miR-135a and miR-135b relative expression were assessed by qPCR. Each value represents the mean ± SD in three independent experiments. *p < 0.05, **p < 0.01, ***p < 0.001, Student’s *t* test. **d** MIA PaCa-2, PANC-1, and BxPc-3 cells were cultured in glutamine-free medium for 24 h. miR-135a and miR-135b relative expression were assessed by qPCR. Each value represents the mean ± SD in three independent experiments. *p < 0.05, **p < 0.01, Student’s *t* test. **e** MIA PaCa-2 cells were cultured in glutamine-free medium for 24 or 48 h. miR-135 expression was assessed by qPCR. Each value represents the mean ± SD in three independent experiments. **p < 0.01, ***p < 0.001, Student’s *t* test. **f** MIA PaCa-2 cells were cultured in glutamine-free medium for 24 h, then glutamine was added back, and cells were cultured an additional 24 h. miR-135a and miR-135b relative expression were assessed by qPCR. Each value represents the mean ± SD in three independent experiments. *p < 0.05, **p < 0.01, ***p < 0.001, Student’s *t* test (Ctrl: complete medium; No Gln: glutamine-free medium). PDAC: pancreatic ductal adenocarcinoma

To identify the mechanism that mediates PDAC adaptation to metabolic stress, we first examined miRNA expression levels in seven pairs of human pancreatic cancer patient tumor tissue along with adjacent normal tissue by RNA-sequencing. miR-135b is the top significantly overexpressed miRNA in tumor tissues (*P* = 0.024, Student’s *t* test) (Fig. 1a). Since the mature forms of miR-135a and miR-135b differ by only one nucleotide and it is hard to distinguish miR-135a and miR-135b (Fig. 1b), we wondered whether this upregulation of both miR-135a and miR-135b exists in human PDAC tumors. To confirm this, we measured the expression of miR-135a and miR-135b in nine pairs of pancreatic patient tumors along with adjacent normal tissue by qPCR. Both miR-135a and miR-135b were highly expressed in PDAC tumors (Fig. 1b), indicating that the miR-135 family is induced in PDAC tumors.

To test if accumulation of miR-135 is due to the nutrient-poor environment of the PDAC tumors, we cultured PDAC cell line MIA PaCa-2 in medium lacking either glutamine, glucose, serine, glycine or cysteine, which were reported to be at low levels in pancreatic tumors^10^. We found the expression of miR-135a and miR-135b only increased in response to glutamine deprivation (Fig. 1c). Interestingly, the increased expression of miR-135b in response to glutamine deprivation was also observed in human fibrosarcoma, breast cancer, and melanoma cell lines (Supplementary Fig. 1). We further tested this using different PDAC cell lines, MIA PaCa-2, PANC-1, and BxPc-3, and found that expression of miR-135a and miR-135b significantly increased in all these cell lines tested upon glutamine deprivation (Fig. 1d). We also measured the expression of miR-135a and miR-135b in MIA PaCa-2 cells cultured in glutamine-free medium for 24 and 48 h. Both miRNAs expression was enhanced with prolonged periods of glutamine deprivation (Fig. 1e). Next, to test whether the increase of miR-135a and miR-135b is reversible, cells were cultured in glutamine-free medium for 24 h, after which glutamine was added back. We found miR-135a and miR-135b expression decreased following the addition of glutamine, suggesting the expression of the miR-135 family is responsive to glutamine stress signaling (Fig. 1f). Taken together, these data suggest that glutamine deprivation induces the expression of miR-135 family in PDAC cells.

### miR-135 is induced by ROS-activated mutant p53

**Fig. 2:**
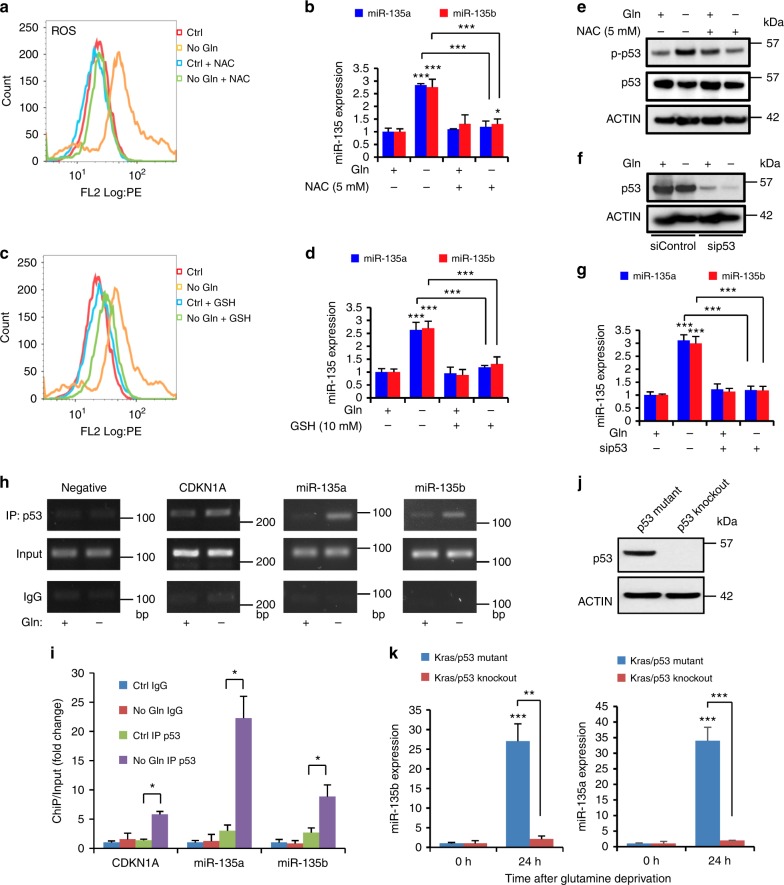
miR-135 is induced by ROS-activated mutant p53. **a**, **c** MIA PaCa-2 cells were cultured in complete medium or glutamine-free medium supplemented with 5 mM NAC or 10 mM GSH for 24 h. Then cells were stained with dihydroethidium and analyzed by flow cytometry or collected to assess relative miR-135a and miR-135b expression by qPCR (**b**, **d**). Each value represents the mean ± SD in three independent experiments. *p < 0.05, ***p < 0.001, Student’s *t* test. **e** MIA PaCa-2 cells were cultured in complete medium or glutamine-free medium supplemented with 5 mM NAC. Phospho-p53 (S15), total p53 and actin were assessed by western blotting. **f** p53 was knocked down by siRNA in MIA PaCa-2 cells. p53 protein was assessed by western blotting. **g** p53 knockdown and control MIA PaCa-2 cells were cultured in complete medium or glutamine-free medium for 24 h. miR-135a and miR-135b expression were assessed by qPCR. Each value represents the mean ± SD in five independent experiments. ***p < 0.001, Student’s *t* test. **h** Chromatin immunoprecipitation analysis was performed to determine p53 binding to the promoters of *CDKN1A*, miR-135a and miR-135b in response to glutamine deprivation. The binding was assessed by DNA gel. **i** The fold change of the p53 binding to the promoters of *CDKN1A*, miR-135a and miR-135b in response to glutamine deprivation were analyzed by qPCR. Each value represents the mean ± SD in three independent experiments. *p < 0.05, **p < 0.01, ***p < 0.001, Student’s *t* test. **j**, **k** Kras/p53 mutant and Kras/p53 knockout mouse PDAC cells were cultured in complete medium or glutamine-free medium for 24 h. Total p53 protein was assessed by western blotting (**j**). miR-135 expression was measured by qPCR (**k**). Each value represents the mean ± SD in four independent experiments. **p < 0.01, ***p < 0.001, Student’s *t* test (Ctrl: complete medium; No Gln: glutamine-free medium). ROS: reactive oxidative species, NAC: *N*-acetyl-l-Cysteine, GSH: glutathione, PDAC: pancreatic ductal adenocarcinoma

Glutamine has been shown to play an important role in controlling reactive oxidative species (ROS)^22^. We asked whether miR-135a and miR-135b are upregulated by the increased ROS experienced during glutamine deprivation. To test this, MIA PaCa-2 cells were cultured in glutamine-free medium supplemented with the antioxidant *N*-acetyl-l-Cysteine (NAC), which can restore the glutathione pool. NAC effectively prevented ROS increase upon glutamine deprivation (Fig. 2a). Glutamine deprivation-induced miR-135a and miR-135b expression were completely inhibited by NAC treatment (Fig. 2b), suggesting that miR-135 family induction is ROS-dependent. Moreover, when MIA PaCa-2 cells were supplemented with the antioxidant glutathione (GSH), the ROS level decreased (Fig. 2c), which was accompanied by inhibition in miR-135 family expression (Fig. 2d). Previously, we showed that glutamine deprivation-induced ROS activates mutant p53 (mutp53)^23^. Similarly, we found that glutamine deprivation-induced mutp53 phosphorylation in MIA PaCa-2 cells, which harbor mutated p53 (Fig. 2e). To assess whether ROS-activated mutp53 contributes to the regulation of the miR-135 family, we knocked down mutp53 in MIA PaCa-2 cells and found that the upregulation of miR-135a and miR-135b upon glutamine deprivation was largely attenuated in p53 knockdown cells (Fig. 2f, g), suggesting mutp53 in pancreatic cancer cells displays a gain-of-function in response to the metabolic stress. To further confirm this, we performed chromatin immunoprecipitation assays (ChIP) in MIA PaCa-2 cells cultured in complete or glutamine-free medium. We found that the binding of mutp53 to the promoters of *CDKN1A*, miR-135a and miR-135b were low in complete medium. However, following glutamine deprivation, mutp53 binding at these promoters was markedly increased (Fig. 2h, i). To further test if the expression of miR-135 is regulated by mutp53 in response to glutamine deprivation, we examined the expression of miR-135a and miR-135b in mouse Kras/p53 mutant and Kras/p53 knockout PDAC cells cultured in glutamine-free medium. Consistent with our finding in human PDAC cells, we found that miR-135 expression was dramatically increased upon glutamine deprivation in Kras/p53 mutant cells. In contrast, this induction was blocked in Kras/p53 knockout cells (Fig. 2j, k). Taken together, these results show glutamine deprivation-induced ROS activates mutp53 and enhances the binding between mutp53 and miR-135 family promoters to increase its expression.

### miR-135 promotes PDAC survival upon glutamine deprivation

**Fig. 3:**
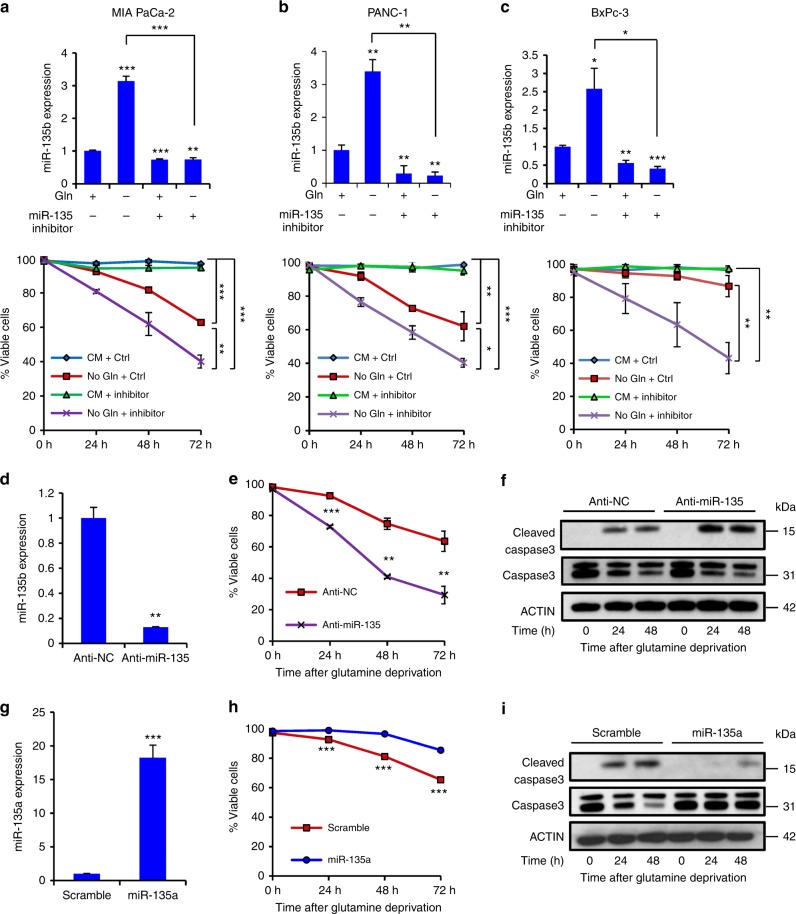
miR-135 promotes PDAC survival upon glutamine deprivation. **a**–**c** MIA PaCa-2 cells (**a**), PANC-1 (**b**), and BxPc-3 (**c**) were transiently transfected with inhibitor control or hsa-miR-135 inhibitor. 48 h posttransfection, cells were cultured in glutamine-free medium. Relative miR-135b expression was assessed by qPCR. Cell viability was assessed by DAPI exclusion flow cytometry at the indicated time points. **d** MIA PaCa-2 cells were transfected with microRNA inhibitor control clone (anti-NC) or miR-135 inhibitor clone (anti-miR-135). miR-135b expression was assessed by qPCR. **e** MIA PaCa-2 cells expressing anti-NC or anti-miR-135 were cultured in glutamine-free medium. Cell viability was assessed by DAPI exclusion flow cytometry at the indicated time points. Cells were also lysed and immunoblotting was performed with indicated antibodies (**f**). Each value represents the mean ± SD in three independent experiments. *p < 0.05, **p < 0.01, ***p < 0.001, Student’s *t* test. **g** MIA PaCa-2 cells were transfected with microRNA scramble control clone or miR-135a precursor clone. miR-135a relative expression was assessed by qPCR. Each value represents the mean ± SD in four independent experiments. ***p < 0.001, Student’s *t* test. **h** MIA PaCa-2 cells expressing scramble or miR-135a were cultured in glutamine-free medium. Cell viability was assessed by DAPI exclusion flow cytometry at the indicated time points. Each value represents the mean ± SD in three independent experiments. ***p < 0.001, Student’s *t* test. **i** Cells were also lysed and immunoblotting was performed with indicated antibodies (CM: complete medium; No Gln: glutamine-free medium). PDAC: pancreatic ductal adenocarcinoma

To determine if miR-135 plays an important role in PDAC cell survival during glutamine deprivation, we transiently transfected human miR-135 inhibitor in MIA PaCa-2, PANC-1 and BxPc-3 cells, and glutamine was removed for 24 h. miR-135a and miR-135b expression was effectively inhibited (Fig. 3a–c and Supplementary Fig. 2a–c). We measured cell viability over time using 4′,6-diamidino-2-phenylindole (DAPI) exclusion flow cytometry. All three PDAC cell lines treated with miR-135 inhibitor were more sensitive to glutamine deprivation (Fig. 3a–c). To further confirm miR-135’s role in survival, MIA PaCa-2 cells were transfected with control vector or an anti-miR-135 construct. Both miR-135a and miR-135b were inhibited in miR-135 knockdown cells (Fig. 3d and Supplementary Fig. 2d). We starved control and miR-135 knockdown cells of glutamine, and measured cell viability over time. miR-135 knockdown cells were more sensitive to glutamine deprivation, which is consistent with our previous results (Fig. 3e). Additionally, increased cleaved-caspase 3, a marker for apoptotic cell death, was observed in these miR-135 knockdown cells cultured in glutamine-free medium for 24 and 48 h compared to control cells (Fig. 3f). Moreover, we used MIA PaCa-2 cells engineered to stably express control vector or miR-135a (Fig. 3g) and starved these cells of glutamine over time. Both cell viability and western blot analysis of cleaved-caspase 3 indicated that miR-135a expressing cells were less sensitive to glutamine deprivation (Fig. 3h, i). Taken together, these data suggest miR-135 family promotes cell survival upon glutamine starvation.

### miR-135 suppresses aerobic glycolysis

**Fig. 4:**
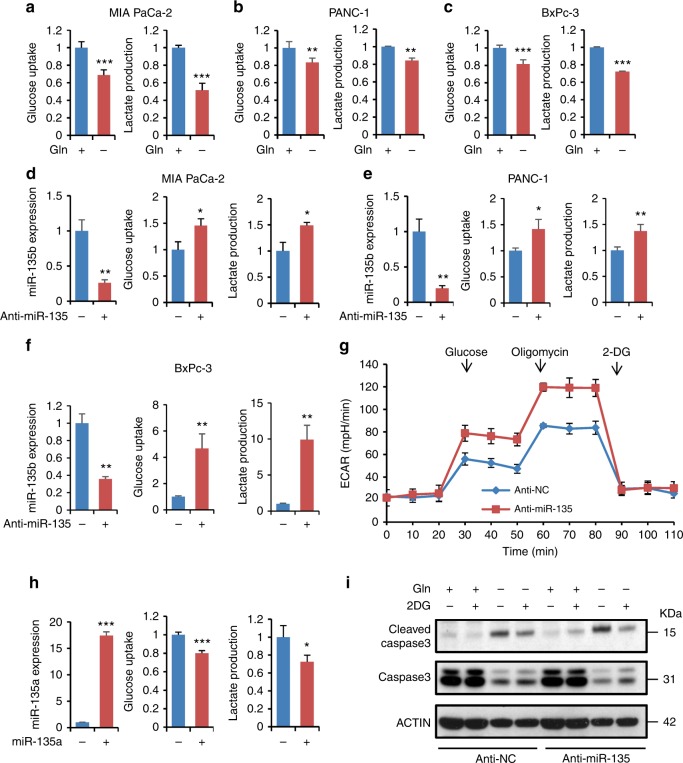
miR-135 suppresses aerobic glycolysis. **a** MIA PaCa-2 cells were cultured in complete medium or glutamine-free medium for 24 h followed by glucose uptake and lactate production measurements using the Nova Bioprofile 100 analyzer. Metabolite levels per cell were normalized to control. Each value represents the mean ± SD in three independent experiments. ***p < 0.001, Student’s *t* test. **b**, **c** PANC-1 cells (**b**) and BxPc-3 cells (**c**) were cultured in complete medium or glutamine-free medium for 24 h followed by glucose uptake and lactate production measurements using the Nova Bioprofile 100 analyzer. Metabolite levels per cell were normalized to control. Each value represents the mean ± SD in four independent experiments. **p < 0.01, ***p < 0.001, Student’s *t* test. **d**−**f** Relative miR-135b expression in MIA PaCa-2 cells (**d**), PANC-1 (**e**), and BxPc-3 (**f**) expressing anti-NC or anti-miR-135 was assessed by qPCR. Cells were cultured in complete medium for 24 h followed by glucose uptake and lactate production measurements using the Nova Bioprofile 100 analyzer. Each value represents the mean ± SD in three independent experiments. *p < 0.05, **p < 0.01, Student’s *t* test. **g** ECAR was measured in MIA PaCa-2 cells expressing anti-NC or anti-miR-135 using the XF-24 Seahorse system. Each value represents the mean ± SD in five independent experiments. **h** Scramble and miR-135a expressing MIA PaCa-2 cells were cultured in complete medium for 24 h followed by glucose uptake and lactate production measurements using the Nova Bioprofile 100 analyzer. miR-135 expression was assessed by qPCR. For qPCR, each value represents the mean ± SD in four independent experiments. ***p < 0.001, Student’s *t* test. For glucose uptake and lactate production, each value represents the mean ± SD in three independent experiments. *p < 0.05, ***p < 0.001, Student’s *t* test. **i** MIA PaCa-2 cells expressing anti-NC or anti-miR-135 were cultured in complete medium or glutamine-free medium supplemented with 2.5 mM 2DG. Cells were lysed and immunoblotting was performed with indicated antibodies

During periods of limited glutamine availability, we have previously demonstrated that a decrease in glycolysis is critical for cell survival^24^. We hypothesized, in pancreatic tumors, miR-135 plays a role in regulating glycolysis during glutamine deprivation. To test this, we cultured MIA PaCa-2, PANC-1, and BxPc-3 cells in complete and glutamine-free medium for 24 h and confirmed glutamine deprivation decreased glucose uptake and lactate production in PDAC cells (Fig. 4a–c). To test if glutamine deprivation-induced miR-135 inhibits aerobic glycolysis, we measured glucose and lactate in medium collected from MIA PaCa-2, PANC-1 and BxPc-3 cells expressing anti-NC or anti-miR-135. Accordingly, glycolysis was significantly upregulated in miR-135 knockdown cells (Fig. 4d–f). In addition, we assessed the glycolytic output during miR-135 inhibition in MIA PaCa-2 cells cultured in glutamine-free medium. In glutamine-deprived state, miR-135 inhibition still led to upregulation of glycolysis (Supplementary Fig. 3a). We next measured the extracellular acidification rate (ECAR), a readout of lactate production, in miR-135 knockdown MIA PaCa-2 cells and found increased glycolytic flux (Fig. 4g); however, miR-135 inhibition did not have a significant effect on oxidative phosphorylation (OXPHOS) (Supplementary Fig. 3b). Moreover, we found glucose uptake and lactate production were significantly decreased in miR-135 overexpressing MIA PaCa-2 cells, indicative of reduced glycolysis (Fig. 4h). In addition, we found that BxPc-3 cells, which endogenously express a higher level of miR-135 correlating with decreased glycolysis, compared to MIA PaCa-2 cells (Supplementary Fig. 3c).

To further test whether miR-135 promotes PDAC cell survival upon glutamine deprivation via glycolysis inhibition, MIA PaCa-2 cells were treated with the hexokinase inhibitor, 2-deoxy-d-glucose (2DG), which suppresses cellular glycolysis (Supplementary Fig. 3d). We found 2DG supplementation sufficiently rescued miR-135 knockdown cells, indicating that an inhibited glycolytic pathway is required for the survival of miR-135 knockdown cells (Fig. 4i and Supplementary Fig. 3e). To further confirm blockade of glycolysis rescues the effect of miR-135 inhibition, we cultured MIA PaCa-2 cells in medium with low glucose and glutamine-free medium. We found low glucose (5 mM) rescued the effect of miR-135 inhibition in glutamine-free conditions (Supplementary Fig. 3f). Taken together, these data suggest miR-135 downregulates glycolysis to promote PDAC cell survival during glutamine deprivation.

### PFK1 is the direct target of miR-135

**Fig. 5:**
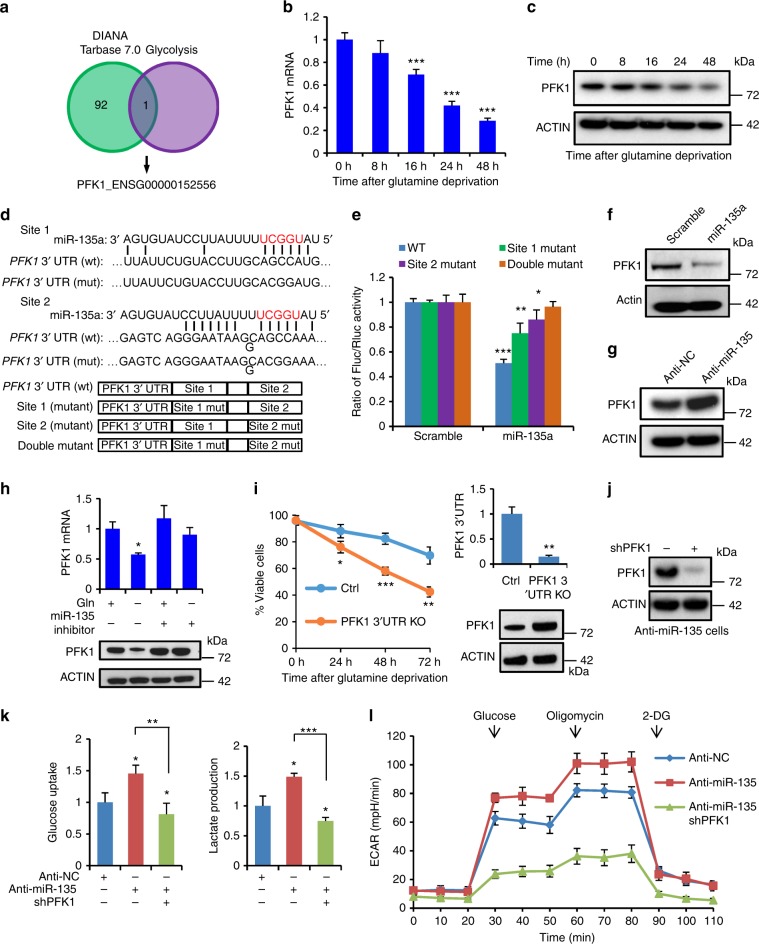
PFK1 is the direct target of miR-135. **a** Overlap of Diana TarBase 7.0 database and Glycolysis enzymes. **b** MIA PaCa-2 cells were cultured in glutamine-free medium, *PFK1* mRNA expression was assessed by qPCR. Each value represents the mean ± SD in three independent experiments. ***p < 0.001, Student’s *t* test. **c** PFK1 protein was assessed by western blotting at the indicated time points. **d** The predicted miR-135a binding sites in the 3′UTR of the human *PFK1* gene, with the corresponding sequence in the mutated (mut) version. Below, schematic of the *PFK1* miR-135-binding site reporter constructs. **e** Dual luciferase assay in MIA PaCa-2 cells expressing scramble or miR-135a and transfected with the reporter constructs from **d**. Each value represents the mean ± SD in four independent experiments. *p < 0.05, **p < 0.01, ***p < 0.001, Student’s *t* test. **f**, **g** PFK1 protein expression was measured in scramble and miR-135a expressing MIA PaCa-2 cells or MIA PaCa-2 cells expressing anti-NC or anti-miR-135 (**g**). **h** MIA PaCa-2 cells were transfected with inhibitor control or miR-135 inhibitor and cultured in complete medium or glutamine-free medium. *PFK1* mRNA was assessed by qPCR and protein was assessed by western blotting. **i** PFK1 3′UTR was knocked out in MIA PaCa-2 cells by CRISPR-Cas9. Cell viability was assessed by Trypan blue exclusion. The 3′UTR expression was assessed by qPCR and the protein level of PFK1 was assessed by western blotting in *PFK1* 3′UTR knockout cells. Each value represents the mean ± SD in three independent experiments. *p < 0.05, **p < 0.01, ***p < 0.001, Student’s *t* test. **j** PFK1 was knocked down in MIA PaCa-2 cells by shRNA. Protein was detected by western blotting. **k** Control (anti-NC), miR-135 knockdown (anti-miR-135) and miR-135/PFK1 double knockdown MIA PaCa-2 cells were cultured in complete medium for 24 h. Glucose uptake and lactate production were measured with the Nova Bioprofile 100 analyzer. Each value represents the mean ± SD in three independent experiments. *p < 0.05, **p < 0.01, ***p < 0.001, Student’s *t* test. **l** ECAR was measured in Control (anti-NC), miR-135 knockdown (anti-miR-135) and miR-135/PFK1 double knockdown MIA PaCa-2 cells using the XF-24 Seahorse system. Each value represents the mean ± SD in five independent experiments

To identify downstream targets of miR-135, we checked previously validated targets in the DIANA TarBase 7.0 database. Interestingly, we found PFK1 was the only target gene predicted to play a role in the glycolytic pathway (Fig. 5a). To confirm whether PFK1 is a potential target of miR-135, the expression of PFK1 was measured over 48 h of glutamine deprivation in MIA PaCa-2 cells. As predicted, mRNA and protein levels of PFK1 decreased over time during glutamine deprivation (Fig. 5b, c). Two putative miR-135 binding sites exist in the *PFK1* 3′UTR. To verify whether these sites mediate a miR-135 regulatory effect on PFK1 expression, we cloned the wild-type *PFK1* 3′UTR (wt) or a miR-135 binding site mutant (mut) into a luciferase reporter plasmid (Fig. 5d and Supplementary Fig. 4a). We transfected this *PFK1* 3′UTR reporter construct into control and miR-135-expressing MIA PaCa-2 cells. miR-135 decreased the luciferase activity of the wt reporters. However, mutation of either miR-135 binding sites resulted in partial restoration of luciferase activity and complete restoration occurred when both sites were mutated (Fig. 5e). Therefore, these results suggest miR-135 binds to both putative target sites of the *PFK1* 3′UTR.

Furthermore, expression of miR-135 led to a decreased expression of PFK1, whereas inhibition of miR-135 resulted in an elevated PFK1 level, suggesting miR-135 regulates PFK1 expression (Fig. 5f, g). Consistently, glutamine deprivation decreased the protein expression of PFK1 in an miR-135-dependent manner (Fig. 5h). To confirm the survival effect of miR-135 is through downregulation of PFK1, we knocked out the *PFK1* 3′UTR region in MIA PaCa-2 cells using CRISPR-Cas9. In support of our conclusion, we found that deletion of the miR-135 target sites in *PFK1* 3′UTR led to more cell death upon glutamine deprivation (Fig. 5i). To determine whether the increased glycolysis observed in miR-135 knockdown cells (Fig. 4d) was due to increased PFK1 activity, we knocked down PFK1 by shRNA in these miR-135 knockdown cells (Fig. 5j). The increased glucose uptake and lactate production exhibited in the absence of miR-135 was abrogated by knockdown of PFK1 (Fig. 5k). To further confirm this, we measured ECAR in control, miR-135 knockdown and miR-135/PFK1 double knockdown cells. Glycolytic flux was substantially attenuated in the double knockdown cells (Fig. 5l). We previously showed that miR-135 induction upon glutamine deprivation is ROS and mutp53 dependent (Fig. 2b, d, g). To test if PFK1 expression is regulated by ROS, MIA PaCa-2 cells were cultured in glutamine-free medium supplemented with the antioxidants NAC and GSH. We found glutamine starvation led to a decrease in PFK1 level, which was reversed by antioxidant reagents, suggesting glutamine deprivation-induced ROS may regulate PFK1 expression via miR-135 (Supplementary Fig. 4b, c). PFK1 expression was partially restored in cells treated with siRNA targeting p53, suggesting that inhibition of miR-135 by p53 knockdown led to PFK1 upregulation (Supplementary Fig. 4d). Taken together, these data demonstrate miR-135 targets PFK1 to decrease glycolytic flux during glutamine deprivation in PDAC cells.

### miR-135 knockdown leads to increased glutamine dependence

**Fig. 6:**
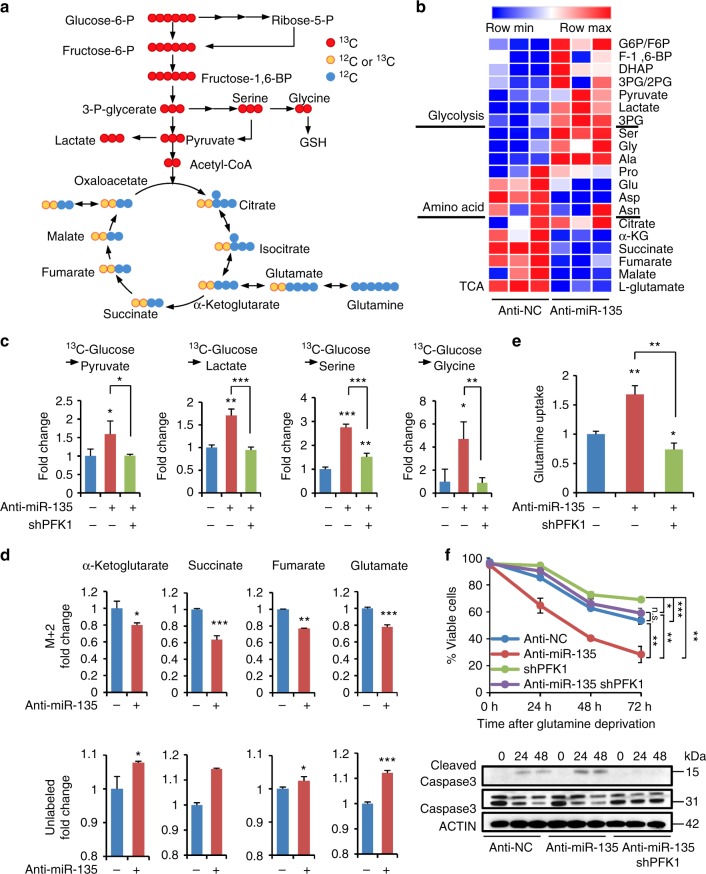
miR-135 knockdown leads to increased glutamine dependence. **a** Schematic representation of ^13^C- glucose metabolism. **b** Heatmap of significantly changed labeled metabolites in glycolysis, amino acids and tricarboxylic acid cycle in control and miR-135 knockdown MIA PaCa-2 cells analyzed by LC-MS. **c** Labeled pyruvate, lactate, serine and glycine were analyzed in control (anti-NC), miR-135 knockdown (anti-miR-135) and miR-135/PFK1 double knockdown MIA PaCa-2. Fold change is calculated by the labeled metabolites normalized to control MIA PaCa-2 cells. Each value represents mean ± SD in three independent experiments. *p < 0.05, **p < 0.01, ***p < 0.001, Student’s *t* test. **d** Labeled and unlabeled α-ketoglutarate, succinate, fumarate, and glutamate were analyzed in control (anti-NC) and miR-135 knockdown (anti-miR-135) MIA PaCa-2 cells by using LC-MS. Fold change is calculated by the labeled or unlabeled metabolites divided by pool and normalized to control MIA PaCa-2 cells. Metabolite data are shown as mean ± SD in three independent experiments. *p < 0.05, **p < 0.01, ***p < 0.001, one-tailed Student’s *t* test. **e** Control (anti-NC), miR-135 knockdown (anti-miR-135) and miR-135/PFK1 double knockdown MIA PaCa-2 cells were cultured in complete medium for 24 h. Glutamine uptake was measured using the Nova Bioprofile 100 analyzer. **f** Control (anti-NC), miR-135 knockdown (anti-miR-135), shPFK1 and miR-135/PFK1 double knockdown MIA PaCa-2 cells were cultured in glutamine-free medium for indicated time points. Cell viability was assessed by Trypan blue exclusion. Each value represents mean ± SD in three independent experiments. *p < 0.05, **p < 0.01, ***p < 0.001, Student’s *t* test. Cells were lysed and immunoblotting was performed with indicated antibodies

To elucidate how miR-135 affects glycolysis in PDAC cells, we performed stable isotope tracer-labeling experiments by culturing control, miR-135 knockdown, and miR-135/PFK1 double knockdown MIA PaCa-2 cells in complete medium containing universally labeled ^13^C-glucose (Fig. 6a). Metabolites from these cells were extracted and analyzed by LC-MS. The labeling experiments showed a significant increase in the enrichment of glucose into glycolytic metabolites in miR-135 knockdown cells (Fig. 6b), consistent with our previous data (Fig. 4d–f). However, we observed a decline in the contribution of glucose carbon to the TCA cycle and TCA cycle derived amino acids, like aspartate, proline, and asparagine (Fig. 6b). Strikingly, PFK1 knockdown reversed the increase in labeled pyruvate, lactate, serine, and glycine observed in miR-135 knockdown cells (Fig. 6c), suggesting miR-135 regulates glycolysis through PFK1. Interestingly, less glucose-derived pyruvate entered the TCA cycle since key intermediates, such as α-ketoglutarate, succinate, fumarate, and glutamate were significantly decreased in miR-135 knockdown cells (Fig. 6d). Meanwhile, miR-135 knockdown cells displayed an increased level of unlabeled α-ketoglutarate, succinate, fumarate, and glutamate (Fig. 6d), indicating these cells utilize glutamine to replenish these TCA cycle intermediates. To confirm this, we cultured control and miR-135 knockdown cells in complete medium containing universally labeled ^13^C-glutamine. We found ^13^C-glutamine-derived succinate, fumarate, malate, and aspartate were increased in miR-135 knockdown cells (Supplementary Fig. 5a). We next measured glutamine uptake in miR-135 knockdown and miR-135/PFK1 double knockdown cells. Consistently, miR-135 knockdown cells were more dependent on glutamine and this increased glutamine uptake is PFK1-dependent (Fig. 6e). Additionally, knockdown of miR-135 in PANC-1 and BxPc-3 increased glutamine uptake (Supplementary Fig. 5b, c). These results suggest the absence of miR-135 upregulates glycolysis, thereby allowing glutamine to replenish TCA intermediates, thus making them more sensitive to glutamine starvation (Fig. 6f). Taken together, these data show that miR-135 inhibits glycolysis directly by repressing PFK1 levels in response to glutamine deprivation.

### Suppression of miR-135 in PDAC cells represses tumor growth

**Fig. 7:**
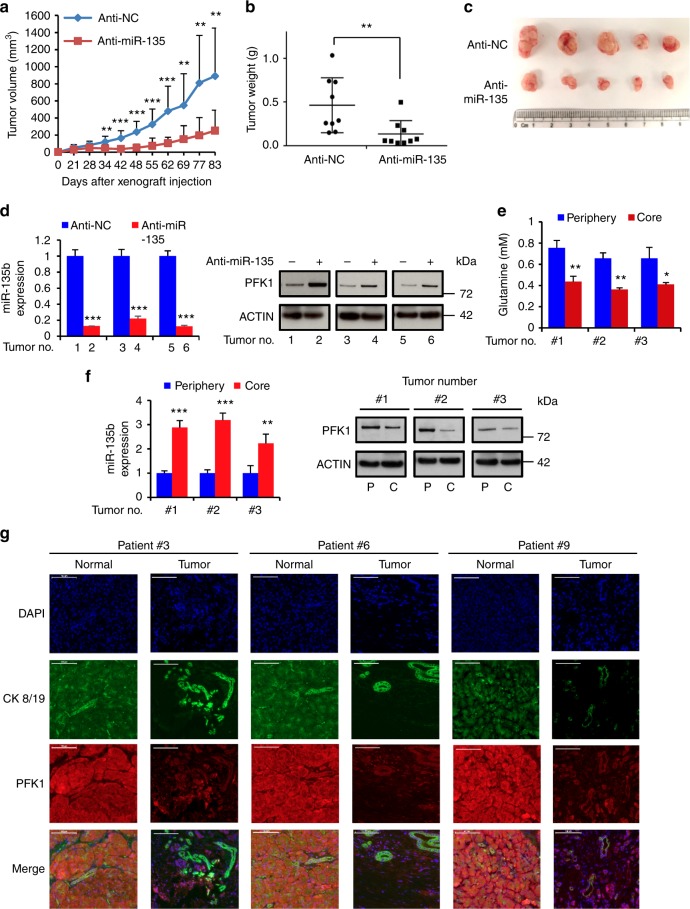
Suppression of miR-135 in PDAC cells represses tumor growth. Nude mice were injected subcutaneously with Control (anti-NC) and miR-135 knockdown (anti-miR-135) MIA PaCa-2 cells. **a** Tumor size was measured over time (*n* = 9 mice per group). **b** Tumor weight was measured after tumors were harvested from mice (*n* = 9 mice per group). Each value represents the mean ± SD. **p < 0.01, ***p < 0.001, Student’s *t* test. **c** Representative tumors from each group. **d** miR-135b expression was determined in xenograft tumors by qPCR and PFK1 protein was assessed by western blotting. **e** Glutamine concentration in samples from peripheral or core regions of control tumors. **f** miR-135b was determined by qPCR and PFK1 protein was measured by western blotting analysis in the core and periphery regions. (C: core; P: periphery). Each value represents mean ± SD in three independent experiments. *p < 0.05, **p < 0.01, ***p < 0.001, Student’s *t* test. **g** Pancreatic patients’ tumor and adjacent normal tissues were stained with DAPI, CK8/19 and PFK1 antibody. Scale bar = 100 µm. PDAC: pancreatic ductal adenocarcinoma

To test whether miR-135 is important for PDAC cell survival in vivo, miR-135 knockdown and control MIA PaCa-2 cells were injected subcutaneously into nude mice. A significant reduction in both tumor growth and weight was observed in miR-135 knockdown tumors (Fig. 7a–c). We checked miR-135 and PFK1 expression in tumors harvested from both groups. As expected, tumors with significantly reduced expression of miR-135 had enhanced levels of PFK1 protein (Fig. 7d and Supplementary Fig. 6a).

To further confirm whether glutamine deprivation induces miR-135 expression in vivo, we evaluated glutamine levels in tumors and serum. We found that compared to the circulating glutamine, PDAC tumors showed significantly decreased glutamine levels (Supplementary Fig. 6b). We dissected the core and periphery regions in control tumors and measured the glutamine concentration in these distinct regions. Levels of glutamine were significantly less in the core versus the periphery regions of these tumors (Fig. 7e), consistent with previous findings^25^. Additionally, we assessed miR-135 expression level (Fig. 7f and Supplementary Fig. 6c) and noticed that miR-135 expression was significantly higher in the core region. Similarly, western blot analysis showed PFK1 was largely reduced in the core compared to the periphery region of the tumors (Fig. 7f). In addition, treatment with 6-Diazo-5-oxo-L-norleucine (DON), a glutamine analog, effectively inhibited tumor growth, confirming previously published data showing PDAC are highly glutamine dependent. Despite the dramatic effect of DON alone, miR-135 inhibition still significantly suppressed tumor growth (Supplementary Fig. 6d). Furthermore, we measured PFK1 expression in human PDAC patients’ normal and tumor tissues by immunohistochemistry. Consistent with miR-135 expression levels (Fig. 1b), PFK1 was decreased in pancreatic tumors (Fig. 7g). Taken together, these data show that inhibition of miR-135 represses pancreatic tumor growth.

## Discussion

Pancreatic cancer is a devastating disease with extremely poor survival rates due to pancreatic tumors exhibiting differential dependency in metabolism^5,26,27^. For example, it has been shown that PDAC cells have increased glutamine metabolism driven by Kras mutations^28^. Moreover, PDAC cells activate salvage pathways such as macropinocytosis to sustain the intracellular requirement of glutamine^13,29^. Thus, due to the selective usage of glutamine, PDAC cells may eventually encounter a glutamine poor environment, which has been supported by a few studies using patient samples. For example, metabolomic analysis of more than 49 patient PDAC samples versus adjacent benign tissues revealed that glutamine levels are significantly decreased in PDAC tumors^10^. Interestingly, a recent effort to use glutaminase inhibitors to treat PDAC showed that pancreatic cancer cells have adaptive metabolic networks that sustain proliferation in vitro and in vivo upon inhibition of glutamine metabolism^30^. Thus, it is important to study the adaptive pathways in PDAC cells when glutamine levels are low, or glutamine metabolism is inhibited.

We found PDAC cells are able to suppress aerobic glycolysis, in response to the low glutamine in the tumor microenvironment, by modulating the expression of miR-135. Recently, the coordination between cellular levels of glutamine metabolism and glycolysis was reported. For example, elevated glutamine levels support cell growth by stimulating aerobic glycolysis^31^, but depletion of glutamine levels in the medium significantly reduces the glycolytic flux^24,32^. In support of this, metabolomic analysis using PDAC patient samples indicate that glycolysis intermediates, including Glycerol-3-Phosphate, Glucose-6-Phosphate, and Fructose-6-Phosphate are significantly decreased in tumors compared with normal tissue^10^. Despite the numerous reports that Kras mutation or hypoxia promote glycolysis, our data suggest other nutritional factors from the tumor microenvironment have an impact on tumor progression that might affect the therapeutic outcome in vivo.

Glutamine contributes to the biosynthesis of glutathione, which neutralizes ROS, and affects the production of NADPH via glutamate dehydrogenase (GLUD). Several studies indicate that glutamine deprivation triggers an increase in ROS levels^22,28,33^. Our previous work and several other groups showed induced ROS led to the activation of mutp53, which displays a gain-of-function feature to adapt to the environment^23,34,35^. Among these target genes of mutp53, miRNAs emerge as important regulators, and play a key role in response to environmental stress^36^. Our ChIP assay showed that, under glutamine deprivation, mutp53 was bound directly to the miR-135 promoter region and induced its expression. Since p53 is frequently mutated in pancreatic cancer, it will be interesting to further investigate how the transactivation of mutp53 is modulated toward microRNAs in response to metabolic stress.

miR-135a and miR-135b are the two isoforms of miR-135. Although they are located at different chromosomes, the mature miR-135a and miR-135b have only one nucleotide difference, which is not in the miRNA binding region. Thus, it is reported that both miRNAs target the same genes^37,38^. miR-135a and miR-135b were previously shown to be critical in cancer tumorigenesis, progression, and metastasis in lung cancer^39–42^. Specifically, miR-135b was identified as an important biomarker in pancreatic cancer^21^. These findings support our hypothesis that miR-135 is significant for pancreatic cancer cells’ ability to resist harsh changes in their microenvironment.

Recently, the coordination between cellular levels of glutamine and glucose was found to be a metabolic checkpoint. Low levels of glutamine, as well as glycolytic intermediates, were observed in pancreatic cancer patient samples compared with normal tissue^10^. Consistently, our data showed that glutamine deprivation upregulated miR-135 expression, which decreased the expression of PFK1 to inhibit glycolysis. Through our metabolomics experiment, we showed that more glutamine entered the TCA cycle in miR-135 knockdown cells, indicating these cells are more dependent on glutamine metabolism. These findings suggest that inhibition of glycolytic flux may decrease glutamine dependence and contribute to PDAC cell survival under low glutamine conditions.

While altered metabolism has long been recognized as a central hallmark of cancer, we have only recently begun to elucidate a mechanistic understanding of PDAC metabolism. Since early attempts to detect or treat PDAC have met with minor success, it is urgent to reveal the features of rewired metabolism in pancreatic cancer with more tractable therapeutic targets. Our results highlight the importance of miR-135 in the posttranslational regulation of PFK1 activity to promote cell survival during glutamine deprivation. Although there is a possibility that miR-135 affects other pathways to promote PDAC cell survival, our data unequivocally establishes a key role for miR-135 in regulating glycolysis in vivo, which is important for PDAC cancer cell adaptation to low glutamine conditions. Interestingly, we found shPFK1 cells displayed a reduced proliferation rate, that suggests a putative role in survival mechanisms (Supplementary Fig. 5d). Therefore, our data highlight the therapeutic potential of targeting miR-135 in combination with glutaminase inhibitors to enhance the treatment of pancreatic cancer.

## Methods

### Reagents and plasmids

Hs_miR-135a Primer (MS00008624), Hs_miR-135b Primer (MS00003472), Hs_RNU6-2_11 Primer (MS00033740), Mm_miR-135a Primer (MS00011130), Mm_miR-135b Primer (MS00001575) and Dual-luciferase reporter assay system (E1910) were purchased from Qiagen. siRNA to p53 (L-003329-00-0005) was purchased from Dharmacon. miRCURY LNA microRNA inhibitor control (199006-001) and hsa-miR-135 microRNA inhibitor (4103932-101) were purchased from Exiqon. Hsa-miR-135a precursor clone in lentiviral (HmiR0052-MR03), miRNA scrambled control clone (CmiR0001-MR03), hsa-miR-135 inhibitor clone in lentiviral (HmiR-AN0170-AM03) and miRNA inhibitor control clones (CmiR-AN0001-AM03) were purchased from GeneCopoeia. TRC Lentiviral Human PFK1 shRNA (RHS4533-EG5213) was purchased from Dharmacon. Kanamycin Sulfate (60615), 2-Deoxy-d-glucose (D8375), Oligomycin A (75351) and NAC (A9165) were purchased from Sigma. Puromycin (P8833), Hygromycin B (H3274) and Lipofectamine RNAiMAX Transfection Reagent (13778075) and Lipofectamine 2000 transfection reagent (11668027) were purchased from Thermo Fisher Scientific.

### Cell culture and transfection

MIA PaCa-2 (CRL-1420^TM^), PANC-1 (CRL-1469^TM^), BxPc-3 (CRL-1687^TM^), HT1080 (CCL-121^TM^), MDA-MB-231 (HTB-26^TM^) and MCF-7 (HTB-22^TM^) were purchased from American Type Culture Collection (ATCC). Patient-derived melanoma M229 was obtained from Roger Lo’s lab (UCLA). Mouse PDAC cell lines derived from primary pancreatic tumors from *LSL-Kras*^G12D^; *p53*^flox/flox^; *Pdx1-CreER* mice treated with tamoxifen to induce oncogenic *Kras*^G12D^ activation and biallelic *p53* inactivation in the pancreas and PDAC cell lines from the *LSL-Kras*^G12D^; *p53*^R172H/WT^; *Pdx1-Cre* (KPC) mouse model were obtained from Tyler Jacks’s lab (MIT)^43^. miR-135a-expressing MIA PaCa-2 cells or miR-135a inhibitor transfected cells were generated via lentiviral mediated transfection. MIA PaCa-2 and PANC-1 were cultured in Dulbecco’s modified Eagle’s medium (DMEM) (10-017-CV, Corning) supplemented with 10% fetal bovine serum (FBS) (FB-12, Omega), 100 units/ml of penicillin, and 100 μg/ml of streptomycin (516106, Sigma). BxPc-3 was cultured in RPMI1640 (10-040-CV, Corning) supplemented with 10% FBS and 100 units/ml of penicillin, and 100 μg/ml of streptomycin. For glutamine starvation experiments, DMEM without glutamine (15-017-CV) or RPMI1640 without glutamine (15-040-CV) was supplemented with 10% dialyzed FBS (FB-07, Omega).

For the treatment with DMEM lacking amino acids, DMEM Ham’s F-12 with HEPES, NaHCO_3_ without amino acids (US Biological D9811-01) was supplemented with 10% dialyzed FBS, and 0.5 mM arginine, 0.5 mM cysteine, 4 mM glutamine, 0.4 mM glycine, 0.3 mM histidine, 0.8 mM isoleucine, 0.8 mM leucine, 1 mM lysine, 0.2 mM methionine, 0.4 mM phenylalanine, 0.4 mM serine, 0.8 mM threonine, 0.08 mM tryptophan, 0.6 mM tyrosine and 0.8 mM valine as complete medium. All the amino acids powder or crystal were purchased from Sigma. To get medium without specific amino acid, DMEM was supplemented with 10% dialyzed FBS and all the amino acids were added except the specified amino acid in figure legends.

To generate lentiviral particles in a 6 cm dish, 293T cells were cotransfected with 1 µg microRNA clone or shRNA vector, 0.5 µg pCMV-VSV-G and 1 µg pCMV-dR8.2 dvpr by Lipofectamine 2000 transfection reagent; viral supernatants were collected after 48 h and 72 h. Cells were infected with the virus supplemented with 10 µg/ml polybrene two times during 48 h. Two days after infection, puromycin or hygromycin B was used to select infected cells. p53 siRNA, hsa-miR-135 microRNA inhibitor and inhibitor control were transfected by Lipofectamine RNAiMAX Transfection Reagent.

### Cell proliferation

Cell proliferation was determined by 3-(4,5-Dimethylthiazol-2-yl)-2,5-Diphenyltetrazolium Bromide (MTT) (M2128, Sigma) according to the manufacturer’s instructions. Briefly, 1 × 10^3^ cells were grown in a final volume of 100 µl culture medium per well of a 96-well plate. At the time of measuring, the medium was replaced by 50 µl of serum-free medium and 50 µl MTT (10 mg/ml) in each well. The plate was incubated at 37 ˚C for 2 h. After incubation, the MTT reagent was aspirated and 100 µl dimethyl sulfoxide (DMSO) was added and the plate was incubated for 10 min. The formation of purple formazan crystals, which are proportional to the number of active cells, was measured using a plate reader (Accuris smartreader 96) at 562 nm.

### Small RNA library preparation and sequencing

Total RNA was extracted using Trizol reagent (15596026, Invitrogen). All libraries were prepared using the Illumina TruSeq Small RNA protocol with minor modification following the manufacturer’s instructions with 12 cycles of PCR amplification after ligation of the 3′ and 5′ adapters. Individual libraries were prepared using a unique index primer in order to allow for pooling of multiple samples prior to sequencing. The library was quantified using qPCR. Sequencing was performed on a Hiseq2500 (Illumina Inc., San Diego, CA) and image processing and base calling were conducted using Illumina’s pipeline. Different miRNA expression analysis was performance on the filtered normalized miRNA count using paired Student’s *t* test. *p* values less than 0.05 were considered significant.

### Quantitative real-time PCR

Total RNA was extracted using Trizol reagent. Reverse transcription reaction was performed using miScript II RT Kit (218161, Qiagen) for miRNAs and qScript cDNA Synthesis Kit (95047-100, Quanta Biosciences) for mRNAs. qRT-PCR was performed in a CFX Connect Real-Time PCR Detection System (Bio-Rad) by using a reaction mixture with SYBR Green PCR Master Mix (95072, Quanta Biosciences). The primers for microRNA were purchased from Qiagen. The primers for *PFK1* mRNA and *18S* RNA are listed in Supplemental Table 1. The PCR cycle parameters were as follows: 95 ˚C for 3 min; 40 cycles with denaturation at 95 ˚C for 10 s, annealing at 55 ˚C for 30 s. All the PCR amplification was performed in triplicate and repeated in three independent experiments. The relative quantities of miR-135a and miR-135b in cells were normalized to U6; the relative quantities of *PFK1* was normalized to human *18S* RNA. The derived values of patient tumor samples were further normalized to those of adjacent normal tissues with the latter being set as 1; the derived values of glutamine deprivation or other amino acids deprivation-treated samples were further normalized to those of complete medium cultured samples with the latter being set as 1; in other cases, the derived values of treated samples were further normalized to those of vehicle-treated samples with the latter being set as 1.

### Immunoblotting

Cells grown in 6 cm dish were lysed on ice in lysis buffer (50 mM Tris-HCL [pH 7.4], 5 mM sodium fluoride, 5 mM sodium pyrophosphate, 1 mM Ethylenediaminetetraacetic acid (EDTA), 1 mM Ethylene glycol-bis(β-aminoethyl ether)-*N*,*N*,*N*′,*N*′-tetraacetic acid (EGTA), 250 mM mannitol, 1% [v/v] Triton X-100) containing protease inhibitor complex (04693159001, Roche). The protein concentrations of the lysates were measured using the BCA Assay kit (23225, Thermo). The lysates were boiled with NuPAGE LDS-PAGE sample buffer (1771559, Invitrogen) supplemented with 5% β-mercaptoethanol (M3148, Sigma) for 5 min. Equal amounts of protein were loaded on precast NuPAGE Bis-Tris Gels (NP0321BOX, Life Technologies) followed by transfer onto nitrocellulose membrane (1620115, Bio-Rad). The immunoblotting was performed with the following antibodies: anti-PFK1 (1:1000) (ab154804, Abcam), anti-β-Actin (1:5000) (A1978, Sigma), anti-caspase 3 (1:1000) (9665s, Cell signaling), anti-cleaved-caspase 3 (1:1000) (9664s, Cell Signaling), anti-p53 (1:200) (sc-126, Santa Cruz), anti-phospho p53 (1:1000) (9284, Cell Signaling), anti-p53 (1:1000) (32532, Cell Signaling).

### CRISPR-Cas9 genomic knockout

*PFK1* 3′UTR was generated using lentiviral CRISPR/Cas9^44^. Briefly, two oligos were designed to target the human *PFK1* 3′UTR gene, using the online design tool at http://crispor.tefor.net/crispor.py. After lentiCRISPR v2 encoding Cas9 (52961, Addgene) was digested with *BsmB*I (R0580S, Biolabs) at 37 ˚C for 30 min, pLentiCRISPR was purified by DNA gel. The two oligos were phosphorylated and annealed at 37 ˚C for 30 min, then ligated with *BsmB*I digested plasmid. MIA PaCa-2 cells were transfected with the lentiCRISPR v2-*PFK1* 3′UTR-specific oligos via lentiviral-mediated transfection and selected by puromycin selection. The oligos to target *PFK1* 3′UTR and primers for testing are listed in Supplementary Table 1.

### Chromatin immunoprecipitation (ChIP) assay

The ChIP assay was performed^45^. Briefly, cells were cultured in complete medium or glutamine-free medium for 24 h and crosslinked by the addition of 1% formaldehyde solution (F8775, Sigma) for 10 min at room temperature. The reaction was stopped by adding glycine (G8790, Sigma) at a final concentration of 125 mM. DNA was immunoprecipitated with p53 antibody and extracted using ChIP assay kit (17295, Millipore). Briefly, cells were rinsed twice with Phosphate-buffered saline (PBS), harvested by a silicon scraper, lysed in ice-old lysis buffer and sonicated to solubilize and shear crosslinked DNA to average size of 200−1000 bp fragments. After centrifugation, the supernatant was transferred to a new tube and diluted with ChIP Dilution Buffer containing protease inhibitors. To reduce nonspecific background, we precleared the diluted cell supernatant with protein A agarose beads for 30 min at 4 ˚C with agitation. The supernatant fraction was collected, and mixed with p53 antibody (sc-126, Santa Cruz) and incubated overnight at 4 ˚C with rotation. Protein A agarose beads were added and rotated for 1 h at 4 ˚C. The agarose beads were pelleted by gentle centrifugation and washed with low salt immune complex wash buffer, high salt immune complex wash buffer, LiCl immune complex wash buffer and TE buffer. The histone complex from the antibody was eluted by elution buffer. 5 M NaCl were added to the eluates and heated at 65 ˚C for 4 h to reverse histone-DNA crosslinks, followed by addition of 0.5 M EDTA, 1 M Tris-HCl (pH 6.5), and 10 mg/ml Proteinase K. DNA was recovered by phenol/chloroform extraction and ethanol precipitation. PCR was performed using Hotstart Taq DNA polymerase (203205, Qiagen). PCR products were detected using agarose gel electrophoresis. PCR primers for the ChIP assays are listed in Supplementary Table 1. qRT-PCR was also performed with SYBR Green PCR Master Mix (95072, Quanta Biosciences) using a CFX Connect Real-Time PCR Detection System (Bio-Rad). The primers for qPCR are the same as PCR experiment above. The PCR cycle parameters were as follows: 95 ˚C for 3 min; 40 cycles with denaturation at 95 ˚C for 10 s, annealing at 55 ˚C for 30 s. All PCR amplifications were performed in triplicate and repeated in three independent experiments. The precipitated DNA fragments corresponding to specific genes were quantified by qPCR and expressed as percentages of their total input DNA fragments.

### Luciferase assay

*PFK1* 3′UTR wild-type clone in pMirTarget (sc209129) which is a reporter construct with firefly luciferase as a reporter was purchased from OriGene. *PFK1* 3′UTR mutant clone was obtained by mutating *PFK1* 3′UTR using Q5® site-directed mutagenesis kit (E0554S, Biolabs). Both clones were cotransfected with Renilla luciferase gene as control to MIA PaCa-2 cells expressing scramble or miR-135. The luciferase activity was measured by Dual-Luciferase® Reporter Assay System following the manufacturer’s instructions. Briefly, cells were washed with ice-cold PBS twice and lysed by lysis buffer. Ten microliters of lysis samples were transferred to tubes containing LARII and mixed thoroughly. The tubes were placed in the luminometer and the reading was taken. Then, 40 μl stop reagent was added in the previous tubes and the Renilla reading taken in the luminometer. The luciferase/renilla signal ratio was calculated.

### Glucose/glutamine uptake and lactate production measurements

2 × 10^5^ cells were plated on each well of a six-well plate and cultured overnight. For MIA PaCa-2 and PANC-1 cells, the medium was replaced by 1 ml DMEM supplemented with 10% FBS; for BxPc-3 cells, the medium was replaced by 1 ml RPMI1640 supplemented with 10% FBS. After culturing for 24 h, the media were collected, and the cell number was counted by an automated cell counter (Bio-Rad). After cell debris was spun down, 0.8 ml medium was used to measure glucose, glutamine and lactate concentrations by the Nova Biomedical BioProfile 100 with fresh DMEM and fresh RPMI1640 as control. The analysis was as followed: glucose uptake = glucose in the fresh medium (mM) − glucose in cultured medium (mM); glutamine uptake = glutamine in the fresh medium (mM) − glutamine in cultured medium (mM); lactate production = lactate in cultured medium (mM) − lactate in the fresh medium (mM). All metabolite measurements were normalized per cell.

### Seahorse assays

The day before the seahorse assay, 7.5 × 10^4^ cells were seeded into seahorse XF^e^24 microplates (102340, Agilent), and the XF^e^24 cartridge (102340, Agilent) was calibrated in the seahorse prep station (Agilent) overnight. Before the assay, the medium was replaced by 0.5 ml XF base Medium (102353, Agilent) supplemented with 2 mM glutamine (25030081, Gibco). Cells were incubated at 37 ˚C for 1 h in the seahorse prep station. 56 μl glucose (100 mM) (G8270, Sigma), 62 µl oligomycin (10 μM) (75351, Sigma) and 69 µl 2DG (1 M) (D6134, Sigma) were added into the cartridge wells. ECAR and OCR levels were determined using seahorse bioscience XF^e^24 extracellular flux analyzer (Agilent) and each cycle of measurement involved mixing (3 min), waiting (2 min), and measuring (3 min) cycles.

### ^13^C-glucose tracing by LC-MS

For ^13^C-Glucose tracing, 2 × 10^5^ cells were seeded in 6 cm plates containing DMEM supplemented with 10% FBS and cultured overnight. Cells were washed with PBS twice and cultured in glucose-free DMEM (11966025, Gibco) supplemented with 10% dialyzed FBS containing ^13^C-glucose (25 mM; Cambridge Isotope Laboratory). After culturing for 6 h, the medium was aspirated at room temperature. Cells were put on dry ice, and 1 ml 80% methanol/water (HPLC grade) was added. The plate was transferred to −80 °C freezer and left for 15 min to further inactivate enzymes. Cells were removed from the −80 °C freezer and put on dry ice and cells were harvested by a silicon scraper. The whole cell extract was transferred to a tube and centrifuged at 20,000 rcf for 10 min at 4 °C. The supernatant was transferred into tubes and dried by speed vacuum. The samples were prepared and analyzed by LC-MS^46^.

### ^13^C-glutamine tracing by GC-MS

For ^13^C-glutamine tracing, 2 × 10^5^ cells were seeded in 6 cm plates containing DMEM supplemented with 10% FBS and cultured overnight. Cells were washed with PBS twice and cultured in glutamine-free DMEM (15-017-CV, Corning) supplemented with 10% dialyzed FBS containing ^13^C-glutamine (4 mM; Cambridge Isotope Laboratory). After culturing for 24 h, the whole cell extract was prepared and dried using the same protocol as glucose tracing. 50 μl  MOX (10 mg/ml in pyridine, 226904 Sigma) was added and the mixture was incubated at 42 °C for 1 h. After the samples were allowed to cool down, 100 μl TBDMS (394882, Sigma) was added and samples were incubated at 70 ˚C for 1 h. Then samples were transferred to GC vials and analyzed by Agilent 5977B GC-MSD- using HP-5MS UI^47^.

### Cell viability and reactive oxygen species measurement

5 × 10^4^ cells were seeded in 24-well plates and cultured overnight. Then cells were cultured in complete medium or glutamine-free medium for 24 h. Cell were washed twice with PBS and resuspended in PBS containing DAPI (0.2 μg/ml) (D9542, Sigma). After a 30-min incubation, cells were washed with PBS three times and examined by flow cytometry (CyAn ADP analyzer).

5 × 10^4^ cells were seeded in 24-well plates and cultured overnight. Then cells were cultured in complete medium or glutamine-free medium supplemented with 5 mM NAC or 10 mM GSH for 24 h. Cells were washed twice with PBS and resuspended in PBS containing dihydroethidium (5 μM) (D1168, Invitrogen). After a 30-min incubation, cells were washed with PBS three times and examined by flow cytometry (CyAn ADP analyzer).

1 × 10^5^ cells were seeded in 12-well plates and cultured overnight. Then cells were cultured in complete medium or glutamine-free medium. Cell were washed twice with PBS and resuspended in PBS containing Trypan blue. The cell viability was assessed by TC20 Automated Cell Counter (Bio-Rad).

### Mouse xenografts

All studies involving animals were performed according to the Institutional Animal Care and Use Committee (IACUC)-approved protocols at the University of California, Irvine. We have complied with the relevant ethical considerations for animal research overseen by this committee. 2 × 10^6^ MIA PaCa-2 control or miR-135 knockdown cells were resuspended in 200 μl DMEM without FBS or antibiotics and were injected into 8-week-old male NCr Nude mice (Taconic Bioscience) by subcutaneous injection. Tumor size was measured every 2−3 days with calipers over time and tumor volume was calculated using the formula ½(length  ×  width^2^). For DON treatment, nude mice were treated with PBS, or DON (10 mg/kg body weight) by intraperitoneal injection every other day for 2 weeks when control tumor volume was around 200 mm^3^. Tumor size was measured over time. At the end of the experiment, mice were euthanized and tumors were harvested. Samples from core and peripheral regions were dissected for further analysis.

### Glutamine extraction and concentration measurement

Tissues were cut into less than 100 mg pieces and homogenized in 70% ethanol by a Precellys 24 homogenizer. After centrifugation at 13,000 rpm for 10 min in 4 °C, the pellets were collected, evaporated to dry and thoroughly extracted with distilled H_2_O (1 μl H_2_O per mg of tissue). The concentration of glutamine was measured by the EnzyChrom Glutamine Assay Kit (BioAssay Systems) following the manufacturer’s instructions. Briefly, for each standard and sample well (96-well plates), working reagents were prepared by mixing 65 µl Assay Buffer, 1 µl Enzyme A, 1 µl Enzyme B, 2.5 µl NAD and 14 µl MTT. 20 μl of standards or samples were transferred into each well and mixed thoroughly. The mixtures were incubated for 40 min at room temperature and added with 100 µl stop reagent. OD was read by a plate reader at 565 nm.

### Immunohistochemistry of human pancreatic tumor samples

Nine pancreatic cancer patient tumor and adjacent normal samples were obtained from the Rambam biobank with ethical approval by the Institutional Review Board at Rambam Health Care Campus, Israel, and informed consents were obtained from patients. Experiments were performed in compliance with all relevant ethical regulations for work with human samples. The specimens were dissected to small sections and only those that were confirmed microscopically to be tumor or normal tissue were included in the analysis. All slides were verified by pathologists at the Rambam Medical Center Biobank specialized in pancreatic histopathology. Specimens who showed pancreatitis histology were not included in the analysis; thus, there were no specimens with an evidence of pancreatitis in this cohort. A distinction between cancerous, normal and pancreatitis tissue was performed by a standard H&E staining technique^48^.

50 μm paraffin-embedded formalin-fixed slides were deparaffinized with xylene, rehydrated and submitted to heat-mediated antigen retrieval in citrate buffer (pH 6.0). Specimens were blocked and permeabilized with 5% BSA in PBS with 0.02% triton for 60 min. PFK1 antibody (orb39009, Biorbyt) was used at 1:100 dilution and incubated with specimen for 60 min. Specimens were then washed with PBS and incubated with Alexa Fluor®647 (ab150075, Abcam). Slides were then washed with PBS and incubated with anti-keratin 8+18+19 antibody (ab41825, Abcam) overnight at 4 °C. The next morning, slides were washed and incubated with Alexa Fluor®488 (ab150113, Abcam) at room temperature for 30 min. Slides were washed with PBS and stained with DAPI (1 mg/ml) for 10 min and mounted with Fluoroguard mounting medium (SCYTEK). Images were acquired using Zeiss Observer Z1 microscope using Axiovision program. Image analysis was performed as followed: High power fields of ×20 magnification of healthy pancreas and tumorous pancreas tissues were captured. The cytokeratin staining was used to discriminate healthy pancreas from PDAC.

### Statistics

For all these experiments, each was repeated independently at least two times with similar results. Results are shown as means; error bars represent standard deviation (SD). The unpaired two-tailed Student’s *t* test was used to determine the statistical significance of differences between means (*p < 0.05, **p < 0.01, ***p < ,0.001) unless indicated separately.

### Reporting Summary

Further information on experimental design is available in the Nature Research Reporting Summary linked to this Article.

## Supplementary Information

Supplementary Information

Reporting Summary

## Data Availability

The miRNA-seq data generated and analyzed in this study are available at the Gene Expression Omnibus (GEO) repository of the National Center for Biotechnology Information under accession code (GSE125538). The authors declare that all data generated from this study are included in this publication and its Supplementary Information or available from the corresponding author on request.
