# Supplementary material for: MiR-135 suppresses glycolysis and promotes pancreatic cancer cell adaptation to metabolic stress by targeting phosphofructokinase-1
Source: Nat Commun. 2019 Feb 18;10:809. doi: 10.1038/s41467-019-08759-0 (PMC6379428; doi:10.1038/s41467-019-08759-0)
Supplement: Supplementary file 1 — Supplementary Information [file 41467_2019_8759_MOESM1_ESM.pdf]

Supplementary Information

MiR-135 suppresses glycolysis and promotes pancreatic cancer cell adaptation to metabolic stress by targeting phosphofructokinase-1

Yang et al.

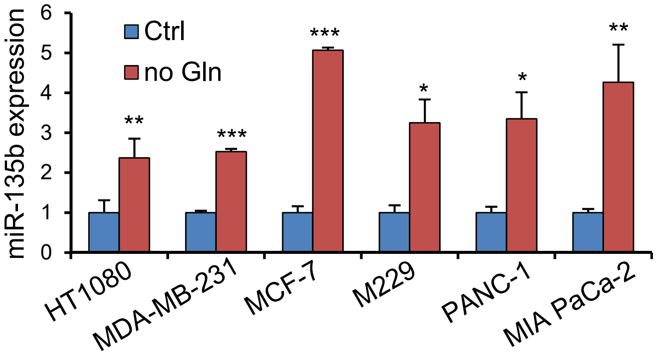

Supplementary Figure 1. miR-135b is induced upon glutamine deprivation in different cell lines. HT1080, MDA-MB-231, MCF-7, M229, PANC-1 and MIA PaCa-2 cells were cultured in complete or glutamine-free medium for 24 hours. miR-135b relative expression was assessed by qPCR. Each value represents mean ±SD in three independent experiments. **p*<0.05, ***p*<0.01, ****p*<0.001, Student's *t*-test. (Ctrl: Complete medium; no Gln: Glutamine-free medium).

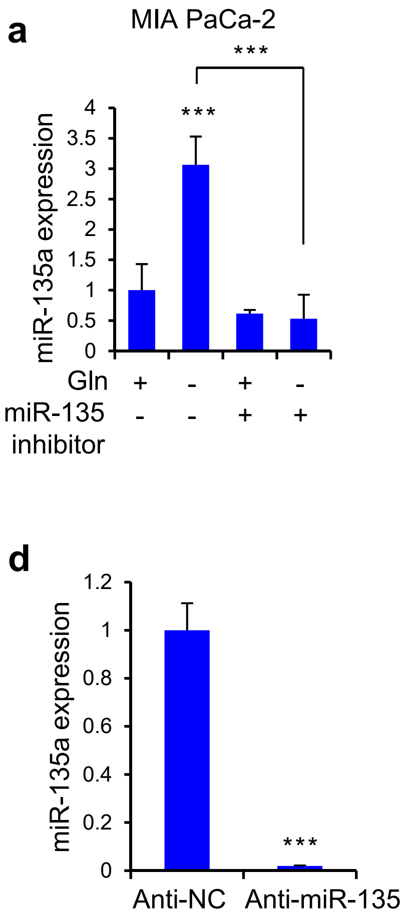

Supplementary Figure 2. miR-135 is required for cell survival under no glutamine. (**a**-**c**) MIA PaCa-2 (a), PANC-1 (b), and BxPc-3 (c) cells were transiently transfected with inhibitor control or hsa-miR-135 inhibitor. 48 hours post-transfection, cells were cultured in glutamine-free medium. Relative miR-135a expression was assessed by qPCR. **d** MIA PaCa-2 cells were transfected with miRNA inhibitor control clone (anti-NC) or miR-135 inhibitor clone (anti-miR-135). miR-135a expression was assessed by qPCR. Each value represents mean ±SD in three independent experiments. *$p<0.05$, **$p<0.01$, ***$p<0.001$, Student's *t*-test.

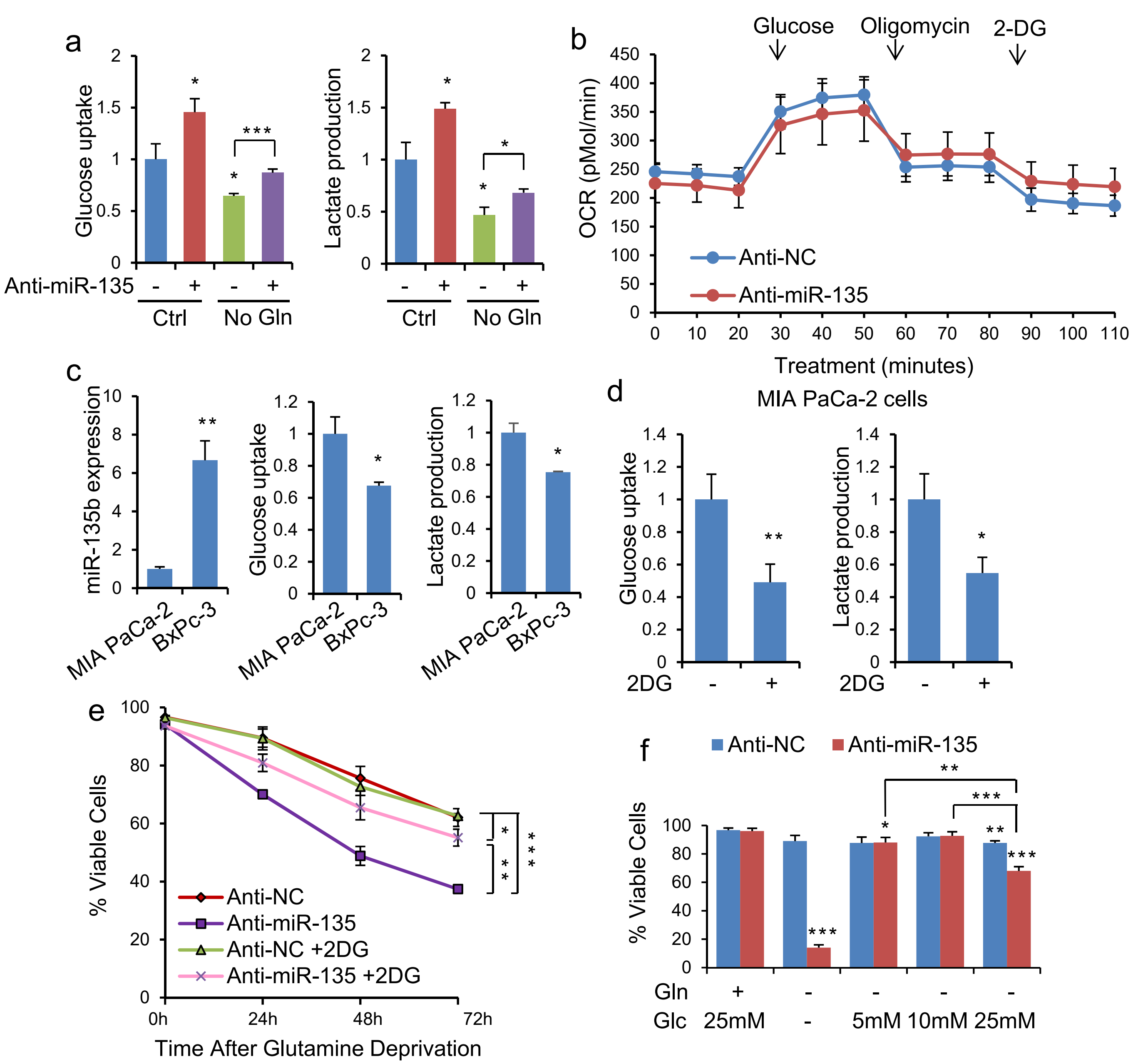

Supplementary Figure 3. miR-135 suppresses aerobic glycolysis. **a** MIA PaCa-2 cells expressing anti-NC or anti-miR-135 were cultured in complete medium (CM) or glutamine-free medium (No Gln) for 24 hours followed by glucose uptake and lactate production measurements using the Nova Bioprofile 100 analyzer. Each value represents mean ±SD in three independent experiments. *p<0.05, ***p<0.001, Student's t-test. **b** OCR was measured in MIA PaCa-2 cells expressing anti-NC or anti-miR-135 using the XF-24 Seahorse system. Each value represents mean ±SD in five independent experiments. **c** Basal miR-135b expression in MIA Paca-2 cells and BxPc-3 cells was assessed by qPCR. Cells were cultured in complete medium for 24 hours followed by glucose uptake and lactate production measurements using the Nova Bioprofile 100 analyzer. **d** MIA PaCa-2 cells were cultured in complete medium supplemented with 2.5 mM 2DG for 24 hours followed by glucose uptake and lactate production measurements using the Nova Bioprofile 100 analyzer. **e** MIA PaCa-2 cells expressing anti-NC or anti-miR-135 were cultured in glutamine-free medium supplemented with 2.5 mM 2DG. Cell viability was assessed by DAPI exclusion flow cytometry. **f** MIA PaCa-2 cells expressing anti-NC or anti-miR-135 were cultured in complete medium, glutamine-free medium and glutamine-free low glucose medium for 24 hours. Cell viability was assessed by Trypan blue exclusion. Each value represents mean ±SD in three independent experiments. *p<0.05, **p<0.01, ***p<0.001, Student's t-test.

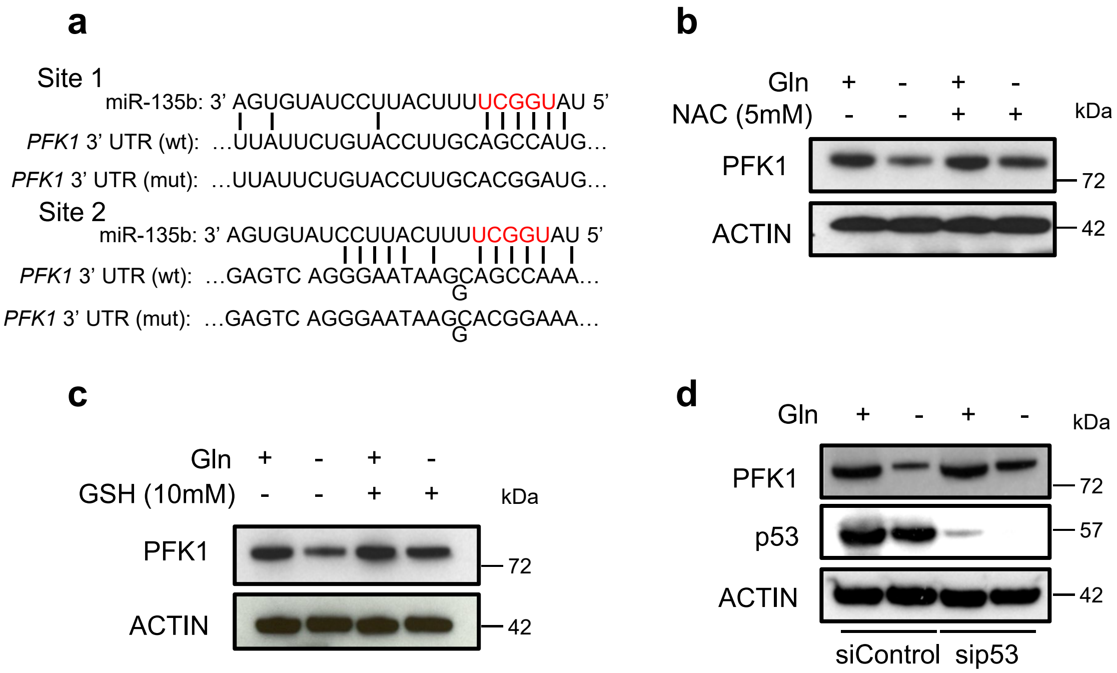

Supplementary Figure 4. PFK1 is regulated by ROS activated mutant p53. **a** The predicted miR-135b binding sites in the 3'UTR of the human *PFK1* gene, with the corresponding sequence in the mutated (mut) version. **b** MIA PaCa-2 cells were cultured in complete medium or glutamine-free medium and supplemented with 5 mM NAC for 48 hours. PFK1 protein expression was assessed by western blotting. **c** MIA PaCa-2 cells were cultured in complete medium or glutamine-free medium and supplemented with 10 mM GSH for 48 hours. PFK1 protein expression was assessed by western blotting. **d** p53 knockdown by siRNA in MIA PaCa-2 cells, which were cultured in complete or glutamine-free medium. PFK1, total p53 and actin protein expression levels were assessed by western blotting.

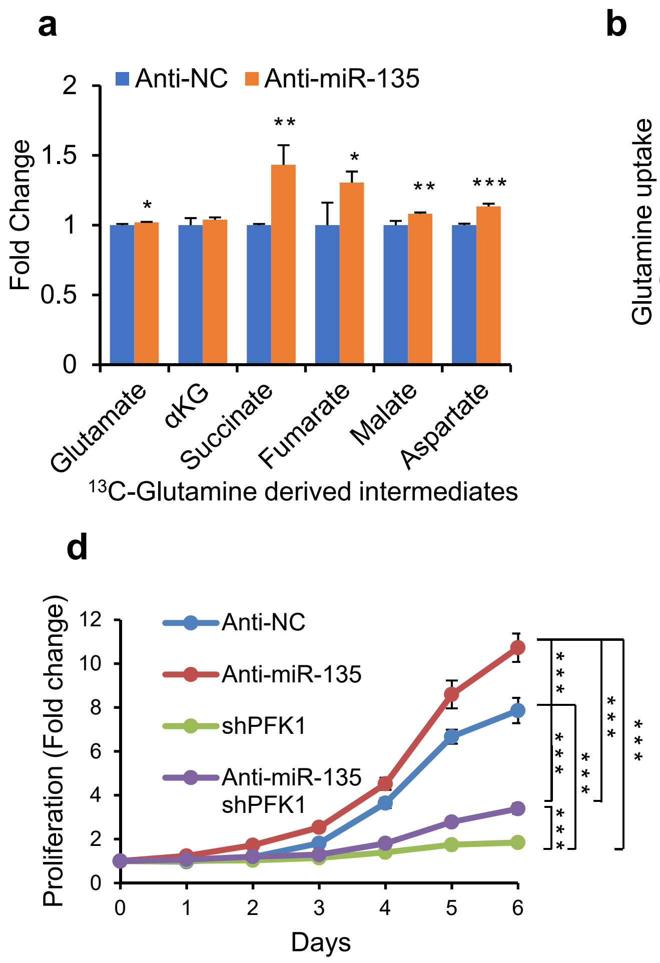

Supplementary Figure 5. Enhanced glycolysis leads to increased glutamine dependence. **a** Control (anti-NC) and miR-135 knockdown (anti-miR-135) MIA PaCa-2 cells were cultured in medium supplemented with ^{13}C- glutamine for 24 hours. Labeled glutamate, α-ketoglutarate, succinate, fumarate, malate, and aspartate were analyzed by using GC-MS. Fold change is calculated by the labeled metabolites divided by pool and normalized to control MIA PaCa-2 cells. **b** Control (anti-NC), miR-135 knockdown (anti-miR-135) PANC-1 cells were cultured in complete medium for 24 hours. Glutamine uptake was measured using the Nova Bioprofile 100 analyzer. **c** Control (anti-NC), miR-135 knockdown (anti-miR-135) BxPc-3 cells were cultured in complete medium for 24 hours. Glutamine uptake was measured using the Nova Bioprofile 100 analyzer. **d** Proliferation of control (anti-NC), miR-135 knockdown (anti-miR-135), shPFK1 and miR-135/PFK1 double knockdown MIA PaCa-2 cells were measured by MTT. Each value represents mean ±SD in three independent experiments. *p<0.05, **p<0.01, ***p<0.001, Student's t-test.

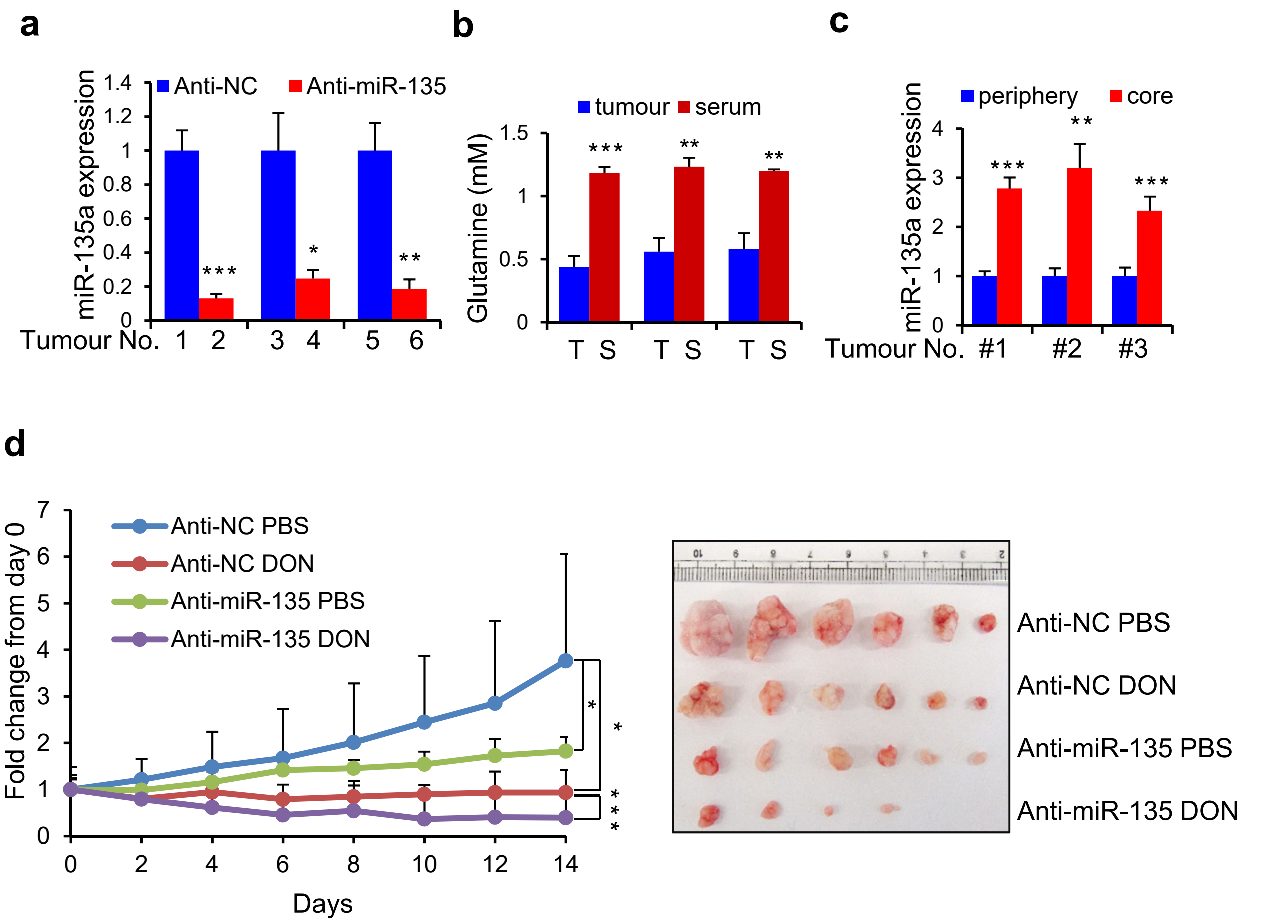

Supplementary Figure 6. Suppression of miR-135 in PDAC cells represses tumour growth. **a** miR-135a expression was determined in Control and miR-135 knockdown MIA PaCa-2 tumours by qPCR. **b** Glutamine concentration in samples from control tumours and serum from healthy nude mice. (T: tumour; S: serum). **c** miR-135a was determined by qPCR in the core and periphery regions. Each value represents mean ±SD in three independent experiments. *p<0.05, **p<0.01, ***p<0.001, Student's t-test. **d** Nude mice were injected subcutaneously with 2 $\times 10^6$ MIA PaCa-2 control or miR-135 knockdown cells. When control tumour size reached an average of 200 mm^3, mice were i.p. injected with PBS or DON (10 mg/kg) every other day for two weeks. (Control tumours treated with PBS, n=6 mice; miR-135 knockdown tumours treated with PBS, n=6 mice; Control tumours treated with DON, n=7 mice; miR-135 knockdown tumours treated with DON, n=6 mice). Tumour size was measured over time. Each value represents mean ±SD, *p<0.05, ***p<0.001, Student's t-test.

Supplementary Figure7. The uncropped images of all blots and gels shown in the major figures of this study.

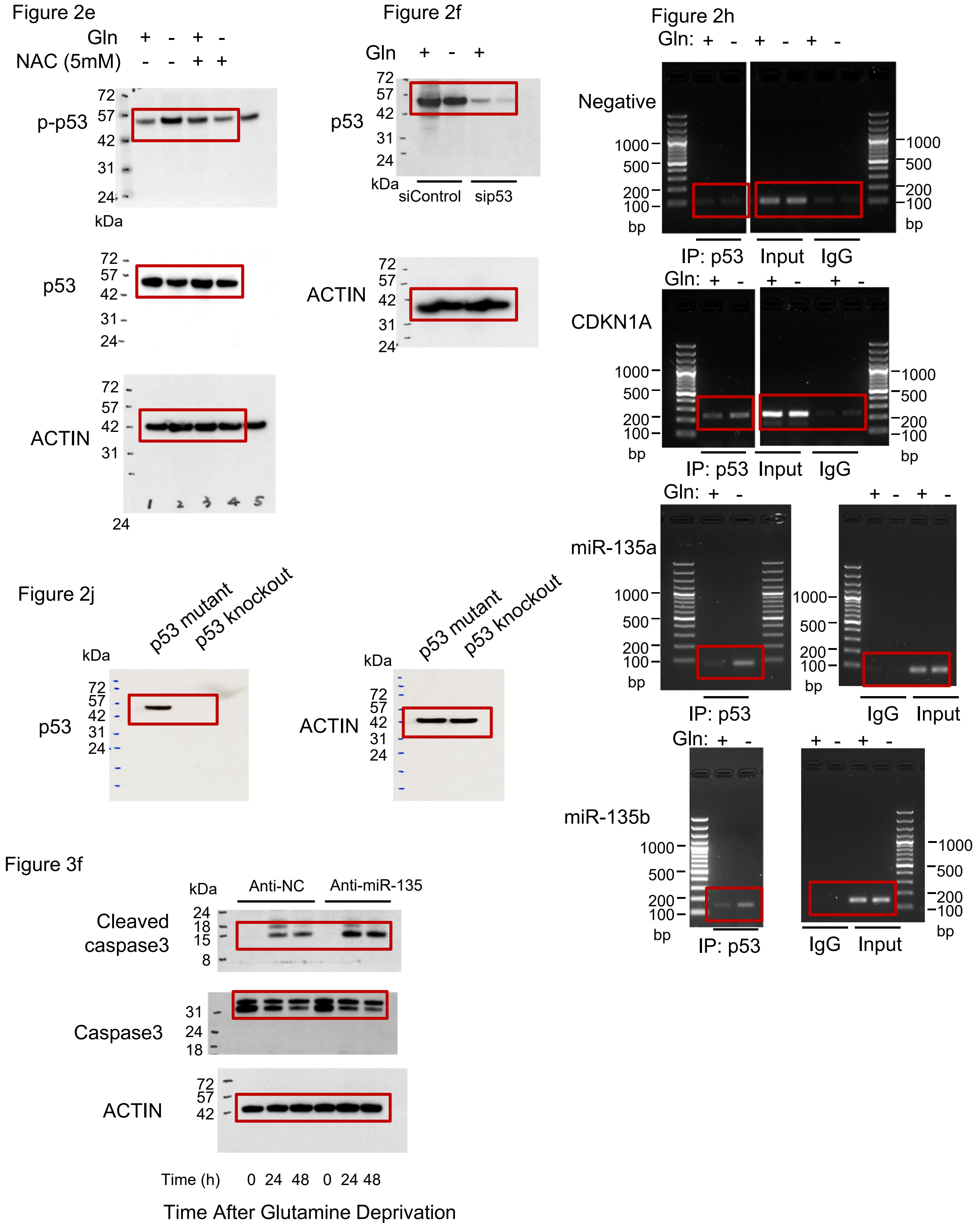

Figure 3i

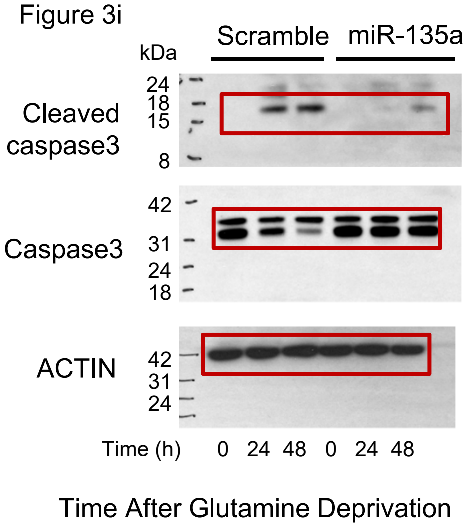

Figure 4i

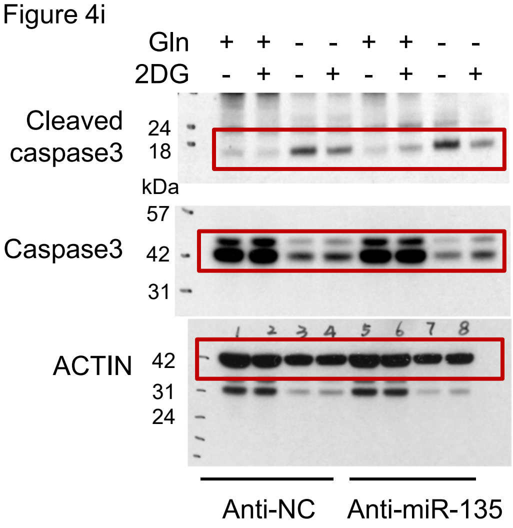

Figure 5c

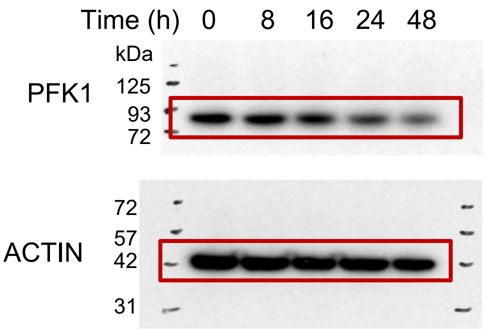

Figure 5f

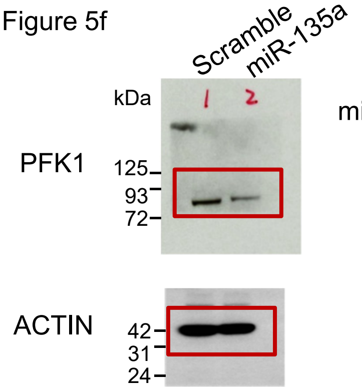

Figure 5h

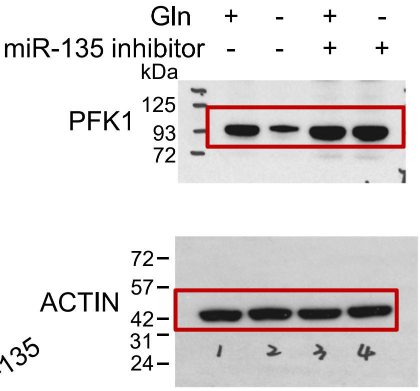

Figure 5g

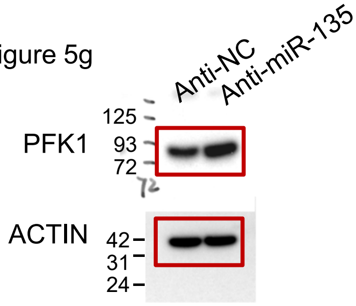

Figure 5i

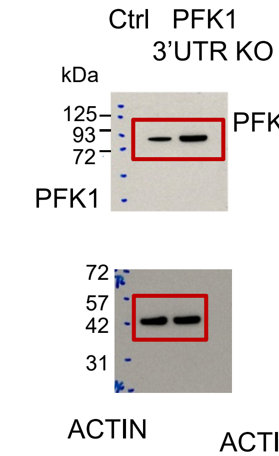

Figure 5j

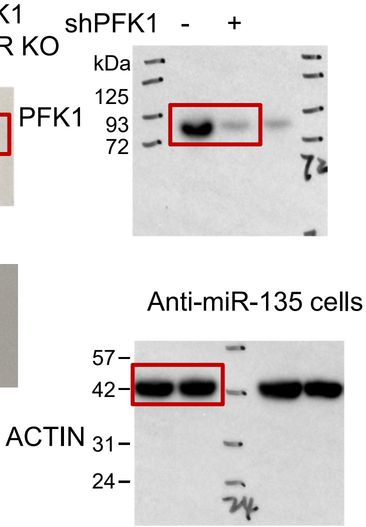

Figure 6f

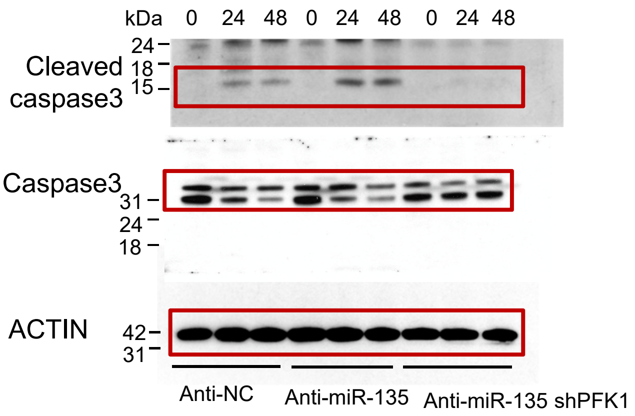

Figure 7d

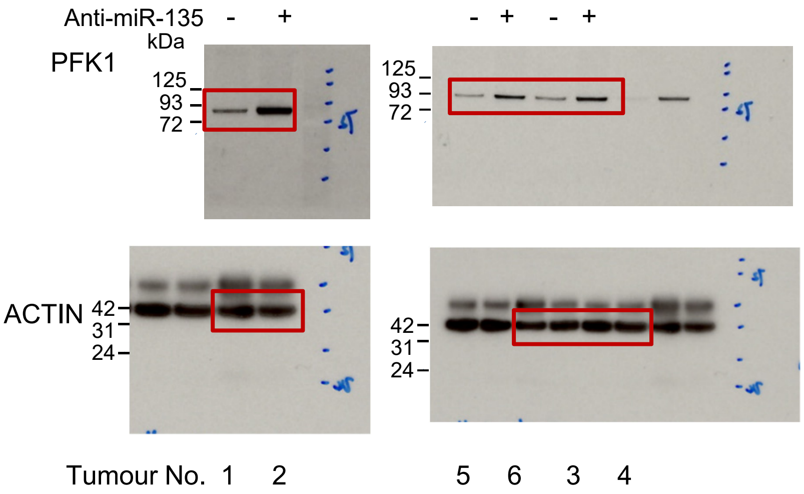

Figure 7f

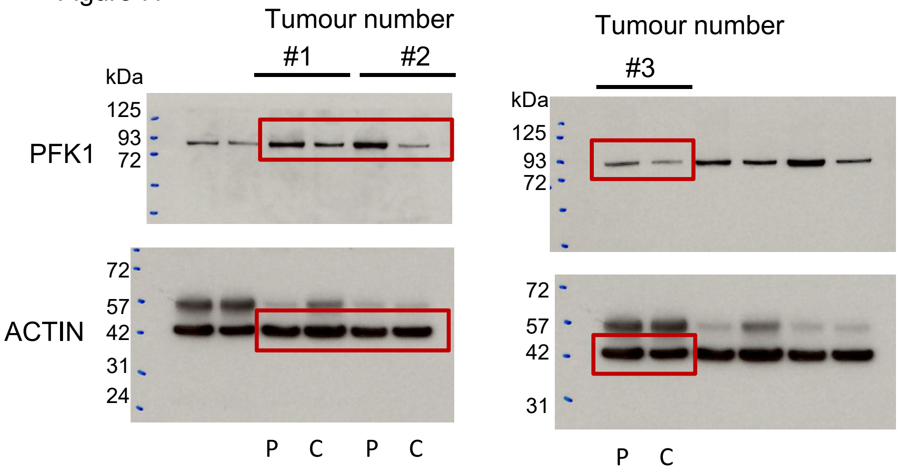

SFigure 3b

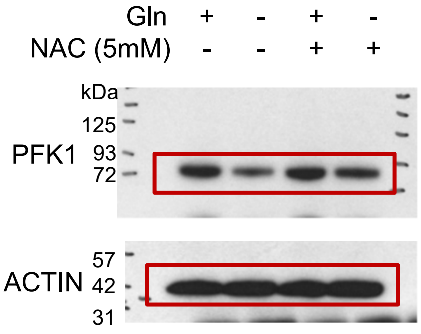

SFigure 3c

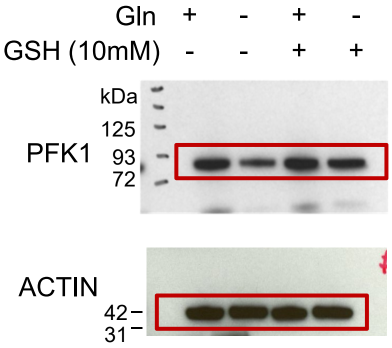

SFigure 3d

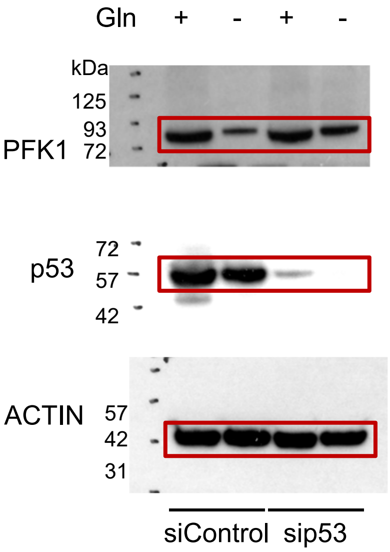

Supplementary Table 1 Sequences of primers used in the study

microRNA	species	Test	Sequence	Catalogue number	Company
miR-135a	Human/m[illegible]	qPCR	5'-UAUGGCUUUUUAUUCCUAUGUGA-3'	MS00008624/MS00011130	Qiagen
miR-135b	Human/m[illegible]	qPCR	5'-UAUGGCUUUUCAUUCCUAUGUGA-3'	MS00003472/MS00001575	Qiagen

Gene	species	Test	Forward	Reverse
PFK1	Human	qPCR	5'- GGTGCCCGTGTCTTCTTTGT-3'	5'-AAGCATCATCGAAACGCTCTC-3'
18s	Human	qPCR	5'-ACCCGTTGAACCCCATTCGTGA-3'	5'-GCCTCACTAAACCATCCAATCGG-3'
PFK1 3'UT[illegible]	Human	qPCR	5'- TACCTTGCAGCCATGACCAG-3'	5'- CCCAGCCTTGTGTGACAGAT-3'
PFK1 3'UT[illegible]	Human	CRISPR-Cas9	5'-CACCGCAGCTTCCCCGGACCGCTTC-3'	5'-AAACGAAGCGGTCCGGGGAAGCTGC-3'
Negative	Human	ChIP	5'-TAAATGGGACAGGTAGGACC-3'	5'- TCCACCGCTTCTTGTCCTGC-3'
CDKN1A	Human	ChIP	5'-GCTGTGGCTCTGATTGGCTTT-3'	5'-ACAGGCAGCCCAAGGACAAA-3'
miR-135a	Human	ChIP	5'- AGCATAAAGTGTGAGGGCAGC -3'	5'- CCCCATGCTGGAGAACACTAA -3'
miR-135b	Human	ChIP	5'- TGCACCTCTCACACTGTCTTC-3'	5'- GTGGTAGGGGGTGAGAAGAGT-3'
