# Supplementary material for: MiR-135 suppresses glycolysis and promotes pancreatic cancer cell adaptation to metabolic stress by targeting phosphofructokinase-1
Source: Nat Commun. 2019 Feb 18;10:809. doi: 10.1038/s41467-019-08759-0 (PMC6379428; doi:10.1038/s41467-019-08759-0)
Supplement: Supplementary file 2 — Reporting Summary [file 41467_2019_8759_MOESM2_ESM.pdf]

nature research

Corresponding author(s): Mei Kong

Last updated by author(s): *YYYY-MM-DD*

Reporting Summary

Nature Research wishes to improve the reproducibility of the work that we publish. This form provides structure for consistency and transparency in reporting. For further information on Nature Research policies, see Authors & Referees and the Editorial Policy Checklist.

Statistics

For all statistical analyses, confirm that the following items are present in the figure legend, table legend, main text, or Methods section.

n/a	Confirmed	
☐	☒	The exact sample size (*n*) for each experimental group/condition, given as a discrete number and unit of measurement
☐	☒	A statement on whether measurements were taken from distinct samples or whether the same sample was measured repeatedly
☐	☒	The statistical test(s) used AND whether they are one- or two-sided<br>*Only common tests should be described solely by name; describe more complex techniques in the Methods section.*
☐	☒	A description of all covariates tested
☐	☒	A description of any assumptions or corrections, such as tests of normality and adjustment for multiple comparisons
☐	☒	A full description of the statistical parameters including central tendency (e.g. means) or other basic estimates (e.g. regression coefficient) AND variation (e.g. standard deviation) or associated estimates of uncertainty (e.g. confidence intervals)
☐	☒	For null hypothesis testing, the test statistic (e.g. F, t, r) with confidence intervals, effect sizes, degrees of freedom and P value noted<br>*Give P values as exact values whenever suitable.*
☒	☐	For Bayesian analysis, information on the choice of priors and Markov chain Monte Carlo settings
☒	☐	For hierarchical and complex designs, identification of the appropriate level for tests and full reporting of outcomes
☒	☐	Estimates of effect sizes (e.g. Cohen's *d*, Pearson's *r*), indicating how they were calculated

Our web collection on statistics for biologists contains articles on many of the points above.

Software and code

Policy information about availability of computer code

Data collection	qPCR-Bio-Rad CFX Maestro 1.0, Illumina Hiseq 2500 System, LC-MS-ThermoScientific,
Data analysis	Microsoft Office Excel 365 ProPlus, GraphPad Prism 7.0, Seahorse Wave Desktop Software, CyAn ADP with Summit Software, LCMS Sieve 2.0

For manuscripts utilizing custom algorithms or software that are central to the research but not yet described in published literature, software must be made available to editors/reviewers. We strongly encourage code deposition in a community repository (e.g. GitHub). See the Nature Research guidelines for submitting code & software for further information.

Data

Policy information about availability of data

All manuscripts must include a data availability statement. This statement should provide the following information, where applicable:

- Accession codes, unique identifiers, or web links for publicly available datasets
- A list of figures that have associated raw data
- A description of any restrictions on data availability

The authors declare that all data generated from this study are included in this publication and its supplementary information files. Source Data are also provided with the online version of the paper. All other datasets are available from the corresponding author upon request.

Field-specific reporting

Please select the one below that is the best fit for your research. If you are not sure, read the appropriate sections before making your selection.

☒ Life sciences ☐ Behavioural & social sciences ☐ Ecological, evolutionary & environmental sciences

For a reference copy of the document with all sections, see nature.com/documents/nr-reporting-summary-flat.pdf

Life sciences study design

All studies must disclose on these points even when the disclosure is negative.

Sample size	For in vitro study, all the experiments were repeated at least three independent times with technical triplicates. For in vivo study, the sample size were estimated based on preliminary data or for statistical power.
Data exclusions	Data exclusion due to technical issues were excluded in these instances: 1. One sample of triplicates were excluded from qPCR experiments if it was clearly an out-lier. 2. For mice experiments treatment with drug, mice which didn't grow tumours were excluded from the experiments.
Replication	All in vitro experiments were repeated at least three independent times with triplicates. For xenograft experiments, 6 or more biologically independent tumours were used.
Randomization	For in vitro study, cells for different groups were treated randomized. For in vivo study, animal injected with control and miRNA knockdown cells were chosen randomly. Animals with xenograft tumours treated with control or glutaminase inhibitor were divided randomly.
Blinding	Cell viablity was conducted by flow cytometry or Trypan blue exclusion counted by a cell counter.

Reporting for specific materials, systems and methods

We require information from authors about some types of materials, experimental systems and methods used in many studies. Here, indicate whether each material, system or method listed is relevant to your study. If you are not sure if a list item applies to your research, read the appropriate section before selecting a response.

Materials & experimental systems

n/a	Involved in the study	
☐	☒	Antibodies
☐	☒	Eukaryotic cell lines
☒	☐	Palaeontology
☐	☒	Animals and other organisms
☒	☐	Human research participants
☒	☐	Clinical data

Methods

n/a	Involved in the study	
☒	☐	ChIP-seq
☐	☒	Flow cytometry
☒	☐	MRI-based neuroimaging

Antibodies

Antibodies used	anti-PFK1, Abcam, ab154804, EPR10734(B)<br>anti-β-Actin, Sigma, A1978, AC-15<br>anti-caspase3, Cell signaling, 9665s, 8G10<br>anti-cleaved-caspase 3,Cell signaling, 9664s, 5A1E<br>anti-p53, Santa Cruz, sc-126, DO-1<br>anti-phospho p53, Cell signaling, 9284<br>anti-p53, Cell signaling, 32532, D2H9O
Validation	Validations are based on the datasheets from the manufacturers.

Eukaryotic cell lines

Policy information about cell lines

Cell line source(s)	MIA PaCa-2 (ATCC®CRL-1420TM), PANC-1 (ATCC®CRL-1469TM), BxPc-3 (ATCC®CRL-1687TM), HT1080 (ATCC®CCL-121TM), MDA-MB-231 (ATCC®HTB-26TM) and MCF-7 (ATCC®HTB-22TM) were purchased from American Type Culture Collection (ATCC). Patient-derived melanoma M229 was obtained from Roger Lo's lab. Mouse PDAC cell lines were obtained from Tyler Jacks's lab.
Authentication	Cells purchased from ATCC were authenticated by ATCC. Patient-derived melanoma M229 was not authenticated.

Mycoplasma contamination	Cells were tested for mycoplasma using MycoAlert mycoplasma detection kit.
Commonly misidentified lines (See ICLAC register)	No

Animals and other organisms

Policy information about studies involving animals; ARRIVE guidelines recommended for reporting animal research

Laboratory animals	NCr Nude mice (Taconic), males, 6 weeks old
Wild animals	No
Field-collected samples	No
Ethics oversight	All studies involving animals were performed according to approved IACUC protocols at the University of California, Irvine

Note that full information on the approval of the study protocol must also be provided in the manuscript.

Flow Cytometry

Plots

Confirm that:

- [x] The axis labels state the marker and fluorochrome used (e.g. CD4-FITC).
- [x] The axis scales are clearly visible. Include numbers along axes only for bottom left plot of group (a 'group' is an analysis of identical markers).
- [x] All plots are contour plots with outliers or pseudocolor plots.
- [ ] A numerical value for number of cells or percentage (with statistics) is provided.

Methodology

Sample preparation	MIA PaCa-2 cells were seeded in 24 well plates and cultured overnight. Then cells were cultured in complete medium or glutamine-free medium for 24h. Cell were washed twice with PBS and resuspended in PBS containing DAPI (0.2 µg/ml) (D9542, Sigma). After a 30 min incubation, cells were washed with PBS 3 times and examined by flow cytometry
Instrument	CyAn ADP analyzer
Software	CyAn ADP with Summit Software
Cell population abundance	All the samples analyzed by flow cytometry were cell lines with different treatments. For each run, 5000 cells were analyzed based on previous experience.
Gating strategy	FSC/SSC gates were done to exclude cell debris. The boundary between positive and negative staining cells was PE>10.

- [ ] Tick this box to confirm that a figure exemplifying the gating strategy is provided in the Supplementary Information.
